# Protein and Microbial Biomarkers in Sputum Discern Acute and Latent Tuberculosis in Investigation of Pastoral Ethiopian Cohort

**DOI:** 10.3389/fcimb.2021.595554

**Published:** 2021-06-04

**Authors:** Milkessa HaileMariam, Yanbao Yu, Harinder Singh, Takele Teklu, Biniam Wondale, Adane Worku, Aboma Zewude, Stephanie Mounaud, Tamara Tsitrin, Mengistu Legesse, Ameni Gobena, Rembert Pieper

**Affiliations:** ^1^ Aklilu Lemma Institute of Pathobiology, Addis Ababa University, Addis Ababa, Ethiopia; ^2^ J. Craig Venter Institute, Rockville, MD, United States; ^3^ Department of Immunology and Molecular Biology, University of Gondar, Gondar, Ethiopia; ^4^ Department of Biology, Arba Minch University, Arba Minch, Ethiopia

**Keywords:** tuberculosis, protein biomarker, sputum, LTBI, microbiome, *Rothia*, acute phase response, α-1-acid glycoprotein

## Abstract

Differential diagnosis of tuberculosis (TB) and latent TB infection (LTBI) remains a public health priority in high TB burden countries. Pulmonary TB is diagnosed by sputum smear microscopy, chest X-rays, and PCR tests for distinct *Mycobacterium tuberculosis* (*Mtb*) genes. Clinical tests to diagnose LTBI rely on immune cell stimulation in blood plasma with TB-specific antigens followed by measurements of interferon-γ concentrations. The latter is an important cytokine for cellular immune responses against *Mtb* in infected lung tissues. Sputum smear microscopy and chest X-rays are not sufficiently sensitive while both PCR and interferon-γ release assays are expensive. Alternative biomarkers for the development of diagnostic tests to discern TB disease states are desirable. This study’s objective was to discover sputum diagnostic biomarker candidates from the analysis of samples from 161 human subjects including TB patients, individuals with LTBI, negative community controls (NCC) from the province South Omo, a pastoral region in Ethiopia. We analyzed 16S rRNA gene-based bacterial taxonomies and proteomic profiles. The sputum microbiota did not reveal statistically significant differences in α-diversity comparing the cohorts. The genus *Mycobacterium*, representing *Mtb*, was only identified for the TB group which also featured reduced abundance of the genus *Rothia* in comparison with the LTBI and NCC groups. *Rothia* is a respiratory tract commensal and may be sensitive to the inflammatory milieu generated by infection with *Mtb*. Proteomic data supported innate immune responses against the pathogen in subjects with pulmonary TB. Ferritin, an iron storage protein released by damaged host cells, was markedly increased in abundance in TB sputum compared to the LTBI and NCC groups, along with the α-1-acid glycoproteins ORM1 and ORM2. These proteins are acute phase reactants and inhibit excessive neutrophil activation. Proteomic data highlight the effector roles of neutrophils in the anti-*Mtb* response which was not observed for LTBI cases. Less abundant in the sputum of the LTBI group, compared to the NCC group, were two immunomodulatory proteins, mitochondrial TSPO and the extracellular ribonuclease T2. If validated, these proteins are of interest as new biomarkers for diagnosis of LTBI.

## Introduction

Diagnosis of active tuberculosis (TB) and latent TB infection (LTBI) is important to control the spread of *Mycobacterium tuberculosis* (*Mtb*), a persistent and increasingly multi-antibiotic drug resistant (MDR) pathogen. TB remains an urgent public health issue in 30 high disease burden countries (World Health Organization, 2015). Eight to 12 million *Mtb* infection cases per year advance to stages with clinical symptoms; 90% of all cases do not cause symptoms (Raviglione et al., 1995). The primary manifestation is pulmonary TB (PTB). Extrapulmonary TB affecting other organs is less prevalent (Smith, 2003). The severity of TB is influenced by intrinsic (genetic) factors (Bellamy et al., 1998; Bellamy et al., 1999; Soborg et al., 2003; Fernando and Britton, 2006) and extrinsic (environmental) factors, such as nutrition and the immunological status of the host. Helminth infections, and particularly HIV/AIDS are comorbidities prevalent in African countries that increase risk of progression to symptomatic TB (Smith, 2003; Potian et al., 2011). The roles of distinct oral and alveolar niche microbiomes as modulators of disease outcome are less well understood (Wu et al., 2013; Adami and Cervantes, 2015). Pertaining to PTB, transmission occurs through inhalation of aerosolized bacteria in droplets.

Certain *Mtb* strains are more transmissible than others (Dye and Williams, 2010), but the reasons for differences in the severity of patient outcomes appear to be linked to their immune responses. *Mtb* invades and manipulates the function of alveolar macrophages, resulting in the formation of granulomas that consist of differentiated epithelioid and multinucleated macrophages, dendritic cells, CD4 and CD8 T-cells as well as B-cells at the infection sites (Tufariello et al., 2003). Key cytokines in the immune response against *Mtb* in the lungs are interferon-γ (IFN-γ) and tumor necrosis factor-α (TNF-α). While IFN-γ is mostly produced by T-cells, TNF-α is released by T-cells and macrophages (Tufariello et al., 2003). Neutrophils are recruited to the infection site and contribute to local inflammation and pathogen containment. In the chronic phase of TB, the balance between Th1 and Th17 responses appears to control bacterial growth and limits the immunopathology. The Th1/Th17 balance was demonstrated in murine TB models while it has not yet been verified for the adaptive immune response to TB in human patients (Torrado and Cooper, 2010). Mycobacterial cells largely reside in the macrophages of granulomas. The latter act as barriers to bacterial spread to other regions of the lungs. Individuals with delayed-type hypersensitivity responses to TB antigens such as ESAT-6 and CFP10, lacking symptoms, are deemed to be latently infected. While not contagious (Tufariello et al., 2003), they are susceptible to disease activation at a later timepoint. An IFN-γ release assay (IGRA) that relies on the stimulation of immune cells in blood plasma with TB antigens followed by the quantitative measurement of secreted IFN-γ is currently the gold standard of LTBI diagnosis (Bastian et al., 2017). Using cytokine antibody arrays, we surveyed the blood plasma of TB, LTBI, and negative community controls (NCC), from the same cohort under study here, and identified RANTES as a potential plasma biomarker of LTBI. This chemokine was differentially abundant with statistical significance (LTBI *vs* TB), with and without prior TB antigen stimulation (Teklu et al., 2018a).

Sputum is a great source of TB biomarkers due to the low health risk associated with sampling and the proximity to the pulmonary infection site. Widely used, but slow or not sufficiently sensitive diagnostic TB tests are acid-fast bacilli (AFB) smear microscopy and the isolation of *Mtb* strains from sputum culture (Ryu, 2015). The PCR-based test Xpert MTB/RIF assay (Cepheid, USA) has higher sensitivity for *Mtb* detection in smear-positive than in smear-negative patients and also detects rifampicin resistance allowing assessment of the antibiotic treatment options (Ryu, 2015). Sputum is also a source of biomarkers that measure inflammation of the lungs in association with other diseases, such as cystic fibrosis (Sagel et al., 2007). Such biomarkers may not be specific for a single respiratory pathogen, irritant, or intrinsic factor. Quantitative changes of metabolites and proteins have been evaluated in clinical samples for TB diagnosis, the latency state, disease progression, and anti-TB treatment responses. Miranda et al. detected sustained levels of ferritin and C-reactive protein (CRP) in patients who remained *Mtb* culture-positive during antibiotic treatments in a Brazilian cohort. These biomarkers indicated persistence of lung inflammation as compared to those who proceeded to a culture-negative state (Miranda et al., 2017). Gopal et al. identified calcium-mobilizing calprotectins as mediators of neutrophil-linked inflammation in granuloma-positive PTB patients and suggested that silencing their activities may attenuate patient symptoms (Gopal et al., 2013). Goletti et al. highlighted the need of developing predictive biomarkers as surrogate endpoints in clinical trials for new investigational anti-TB drugs (Goletti et al., 2016). A neutrophil-driven blood transcriptional signature induced by IFN-γ was identified for active TB (Berry et al., 2010) consistent with neutrophil infiltration of the patients’ infected lungs. Jiang et al. demonstrated reduced expression of CD27 in TB antigen-targeting CD4 T cells during persistent infection and commented on the role of this T-cell surface biomarker for diagnosis of chronic TB (Jiang et al., 2010). Of particular interest are biomarkers that discern LTBI from healthy controls since there is a risk of pathogen reactivation. One study revealed proteomic fingerprints in TB antigen-stimulated plasma of patients and described greater than 82% specificity and 89% sensitivity distinguishing TB from LTBI (Sandhu et al., 2012). A proteomic approach was reported to discern LTBI from healthy controls at greater than 85% specificity and sensitivity using non-stimulated plasma samples (Zhang et al., 2014). Protein biomarkers suggesting protective effects of vaccines are of high clinical interest. Gopal et al. found IL-17 to be involved in protective immunity against a virulent *Mtb* strain using a mouse model of lung infection. Furthermore, a role of IL-17 in driving murine Th1 cell responses upon BCG vaccination was suggested (Gopal et al., 2012; Gopal et al., 2014).

The impact of oral and respiratory tract microbiomes on human health are in an exploratory phase and generally difficult to unravel due to the complexity of microbiome-host relationships (Dewhirst et al., 2010). Distinct respiratory tract-colonizing bacterial taxa may correlate with, cause susceptibility to, or mediate protection from lung diseases including TB. In the context of asthma in infants, such a causal relationship was indeed identified (Teo et al., 2015). Studies of the oral and respiratory tract microbiomes for TB patients have been limited to date, as reviewed by Hong et al. (2016) and Naidoo et al. (2019). Here, our objectives were to investigate an interesting multi-ethnic cohort of Ethiopian pastoralists (such groups are generally neglected in basic research studies due to both geographic remoteness and poverty), to identify quantitative differences in the proteomes and microbiomes of sputum using high-dimensional analysis methods for three cohorts (TB, LTBI, NCC), and to gain additional insights into the host-*Mtb*-microbiome crosstalk. The analyses could result in novel biomarker candidates for TB and LTBI.

## Materials and Methods

### Human Subjects and TB Diagnostic Approaches

Human subject recruitment occurred in a multi-ethnic southern Ethiopian region (South Omo province). Covering eight districts, a study named “Systems Biology for Molecular Analysis of Tuberculosis in Ethiopia” resulted in informed consent of ~2,000 individuals aged 16 years or older at local or regional clinics. Parental consent was obtained for children ranging in age from 12 to 15 years. Some individuals had symptoms consistent with TB. Negative community controls (NCC) often were household contacts of those suspected to be infected with TB. Nearly 1,200 sputum and blood samples were collected. Of those, nearly 13% were positive based on clinical signs and symptoms, AFB smear microscopy, and/or isolation of *Mycobacterium tuberculosis* complex (MTBC) strains on Lowenstein Jensen medium (Wondale et al., 2018). Nearly 500 participants were tested for LTBI employing IFN-γ assays; 50.5% of them were positive for LTBI (Teklu et al., 2018b). Subjects positive for PTB were offered DOTS treatments at a clinic in proximity to their residences (World Health Organization, 1997). Information on the geographic and socio-economic characteristics, ethnic affiliations, exclusion from participation, and other medical data were reported recently (Wondale et al., 2018; Teklu et al., 2018b), a subset of which are included in **Supplementary File S1**. The Institutional Review Board at Aklilu Lemma Institute of Pathobiology, Addis Ababa University (ALIPB-AAU) under ref. no. ALIPB/IRB/22-B/2012/13, the National Research Ethics Committee of Ethiopia (ref. no. 3.10/785/07), and the J. Craig Venter Institute’s IRB (ref. no. 2014-200) approved the human subject protocol. Subjects not diagnosed with TB donated blood samples to perform IRGAs, allowing separation of the LTBI group from healthy individuals based on QuantiFERON-TB Gold In-Tube test (GFT-GIT) data. We set the positivity threshold for LTBI at the recommended value of ≥0.35 IU/ml (Teklu et al., 2018b).

### Sputum Sample Collection and Processing

Study participants were asked to cough up sputum. It was collected in pre-labeled cups. Where possible, subjects completed three cycles of sputum expectoration using 3–5% hypertonic sodium chloride. The single-timepoint specimens were combined and processed as described previously (HaileMariam et al., 2018). Briefly, collected sputum aliquots for proteome and microbiome analysis were disinfected with SDS buffer (1% SDS, 10 mM Na-EDTA, 50 mM DTT, 0.03% Tween-20, 50 mM Tris/Tris−HCl, pH 8.0) and centrifuged at 16,000 × g to recover the supernatants. SDS-denatured and heat-treated lysates (85°C) were shipped to the site of “omics” analyses (J. Craig Venter Institute) and stored long-term at −80°C prior to analyte extraction. Protein samples were run in SDS-PAGE gels (4–12%T) to estimate total protein concentrations. Aliquots of 150 µg protein extract were subjected to S-Trap Ultra-Fast sample preparation (HaileMariam et al., 2018), digesting proteins with trypsin at a 50:1 mass ratio. Peptides were desalted prior to LC-MS/MS analysis using the spinnable StageTip protocol (Yu et al., 2014a). rDNA extraction and analysis are described below.

### Shotgun Proteomics

An Ultimate 3000-nano liquid chromatography (LC) system coupled to a Q-Exactive mass spectrometer (both units from Thermo Scientific) was used for LC-MS/MS analysis. The workflow and data acquisition methods have been described comprehensively (Yu et al., 2014b). Briefly, peptide digestion products of approximately 10 µg were separated over a 150 min gradient from 2 to 80% acetonitrile (120 min to 35%, 10 min to 80%), with 0.1% formic acid in buffers A and B. The flow rate using an in-house packed column (75 µm × 15 cm, 3.0 µm ReproSil-Pur C18-AQ media) was 200 nl/min. MS survey scans were acquired at a resolution of 70,000 over a mass range of m/z 350–1,800. During each cycle in a data-dependent acquisition mode, the 10 most intense ions were subjected to high-energy collisional dissociation (HCD) applying a normalized collision energy of 27%. MS/MS scans were performed at a resolution of 17,500. Two technical replicate LC-MS/MS runs were performed per sample, and the MS data were combined in the MaxQuant analysis process.

### Identification and Quantitation of Proteins

The MS raw data were processed using the Proteome Discoverer platform (version 1.4, Thermo Scientific) and the Sequest HT algorithm. The database contained protein sequences from the Mtb strain ATCC 25618/H37Rv (7,955 entries) and human proteins (20,195 sequence entries; reviewed sequences only; version 2015_06). The search parameters included two missed tryptic cleavages, oxidation (M), protein N-terminal acetylation and deamidation (N, Q) as variable modifications, and carbamidomethylation (C) as a fixed modification. Minimum peptide length was seven amino acids. The MS and MS/MS ion tolerances were set at 10 ppm and 0.02 Da, respectively. The false discovery rate (FDR) was estimated employing the integrated Percolator tool. Only protein hits identified with a 1% FDR threshold were accepted. For protein quantification, the MaxQuant and Andromeda software suite (version 1.4.2.0) was used, accepting most default settings provided in the software tool (Yu et al., 2017a). Label-free quantification (LFQ) generates relative protein abundance data from integrated MS1 peak areas of the high-resolution MS scans (Cox et al., 2014). Only proteins quantified by at least one unique peptide were used for analysis. LFQ values were log (base 2) transformed, and then imputed with respect to missing values. Clustering and correlation analyses were performed using functions embedded in the Perseus (version 1.5.0.15) software. The LC-MS/MS data were deposited in ProteomeXchange *via* the PRIDE partner repository under the dataset identifier is PXD012412.

### 16S rDNA Sequencing

Aliquots of 300 µl of the SDS-denatured sputum lysates were subjected to phenol-chloroform extraction to isolate total DNA. Ethanol-precipitated enriched DNA extracts were subjected to PCR amplification using 515F and 806R forward and reverse primers, respectively, to amplify the V4 region of microbial DNA in the extracts. The PCR amplification method was previously described (Claassen-Weitz et al., 2018). A two-step amplification was performed to reduce the risk of non-specific binding when using adapters/sequencing primers of more than 100 base pairs (bp). For 167 individual specimens, sufficient quantities of the V4 sequence region (254 bp) were amplified as visualized by ethidium bromide staining in agarose gels. Samples for DNA library preparation were obtained by excising bands of approximately 300 bp and normalizing the DNA quantity per sample by quantifying with Quanti-iTTM PicoGreen^®^ (Life Technologies, USA). Amplicons were pooled at 100 ng each. A positive control (*Escherichia coli* DNA extract) was subjected to the same amplification and purification protocol. A standard Illumina sequencing-based library preparation and sequencing protocol (MiSeq Reagent Kit v3, 600 cycles) was used as described (Claassen-Weitz et al., 2018). The library dilution had 15% PhiX as an internal control at a 4 pM concentration.

### Processing and Filtering of 16S Sequence Reads

We generated operational taxonomic units (OTUs) *de novo* from raw Illumina 16S rDNA sequence reads using the UPARSE pipeline (Edgar 2013). Methods for trimming of the adapter sequences, barcodes and primers, the elimination of sequences of low quality, the de-replication step, and sequence read abundance determinations were analogous to those applied previously (Singh et al., 2019). Chimera filtering of the sequences occurred during the clustering step. We used the Wang classifier, bootstrapping using 100 iterations and mothur to report full taxonomies for only those sequences where 80 or more of the 100 iterations were the same (cutoff = 80). The taxonomies were assigned to OTUs using mothur (Schloss et al., 2009) with the version 123 of the SILVA 16S ribosomal RNA database as the reference (Quast et al., 2013). From the tables of OTUs with corresponding taxonomy assignments, we removed likely non-informative OTUs (rare OTUs and taxa strongly affected by MiSeq sequencing errors). Unbiased metadata-independent filtering was applied at each taxonomy level by eliminating features that did not pass the selected criteria (<2,000 reads and OTUs present in less than 10 samples), as described previously (Singh et al., 2019). The 16S rDNA sequencing data were deposited in NCBI BioProject under dataset identifier PRJNA663902.

### Identification of Phylogenetic Groups for Gut and Oral Microbiota

The phyloseq package version 1.16.2 in R package version 3.2.3 was used for the microbiome census data analysis (McMurdie and Holmes, 2013). The plot_richness function was used to create a plot of alpha diversity index estimates for each sample. The differences in microbial richness (α-diversity) were evaluated using Wilcoxon t-test. The ordination analysis was performed using the non-metric multi-dimensional scaling (NMDS) with the Bray–Curtis dissimilarity matrix (Bray, 1957). The data output was used for the generation of a heatmap using the plot_heatmap function including a side bar where clinical variables associated with each sample were assigned to look for specific associations (McMurdie and Holmes, 2013).

### Statistical Analyses of Microbiome and Proteomic Data

To detect differential abundances in microbiota at a genus or species level the DESeq2 package version 1.12.3 in R was used. Phyloseq data are converted into a DESeq2 object using the function phyloseq_to_deseq2 function. DESeq2 (Love et al., 2014) is a method for the differential analysis of count data that uses shrinkage estimation for dispersions and fold changes to improve both stability and interpretability of the estimates. The DESeq2 test uses a negative binomial model rather than simple proportion-based normalization or rarefaction to control for different sequencing depths, which may increase the power and also lower the false positive detection rate (McMurdie and Holmes, 2014). Default options of DESeq2 were used for multiple testing adjustment applying the Benjamini-Hochberg method (Benjamini YH, 1995). To detect differential abundances in proteomic datasets, we used the Welch t-test, an unequal variance two-sample location test embedded in the Perseus software tool. The P-value significance threshold was set at ≤0.01. To identify enriched biological pathways from differentially abundant proteins (TB *vs* LTBI), we employed GO term analysis (http://geneontology.org/). To determine the enriched expression of proteins in tissues or anatomical locations, we reviewed relevant information in the Protein Atlas database (https://proteinatlas.org/).

### Western Blotting

Approximately 10 µl of the sputum lysates were run in 4–12%T SDS-PAGE gels, electroblotted onto PVDF membranes (1.5 h at 150 V), and incubated with antibody dilutions separated by intermittent PBS/0.1% Tween-20 wash steps as reported earlier (Yu et al., 2017b). A primary anti-human α-1-acid glycoprotein (α1AGP) polyclonal IgG fraction antibody developed in rabbit (Sigma-Aldrich; A0534) was used at a 1:2,000 dilution (overnight at 4°C). A secondary goat anti-rabbit IgG-HRP conjugate (Sta. Cruz Biotech; sc-2004) was used at a 1:10,000 dilution (2 h at 20°C). HRP-catalyzed color development with 3,3’-diaminobenzidine (DAB) required circa 5 min of incubation at 20°C.

## Results

### Human Subjects

**Figure 1:**
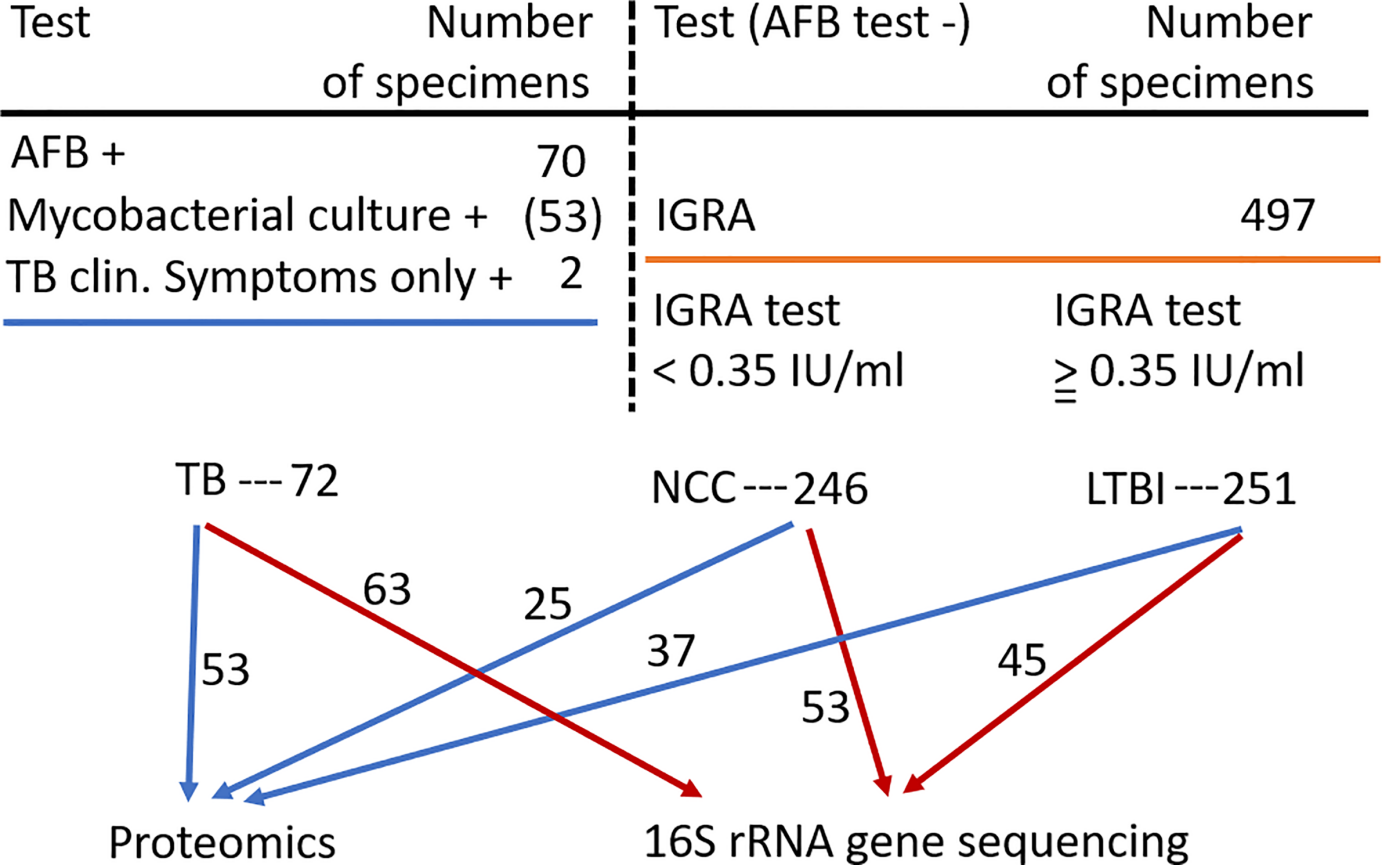
Flow chart showing the three human subject groups and the specimens used for sputum proteomics and 16S rRNA gene sequencing analyses. Abbreviations used are denoted in the text.

Inhabitants of the South Omo province in southern Ethiopia are highly diverse with respect to ethnicity, culture, and language. Many of its people have a traditional lifestyle as cattle herders or in subsistence agriculture with little access to medical care. The major ethnic groups are Hamar, Daasanech, Bena, Tsemay, Selamago, Maale, Ari, and Nyangatom. The prevalences of TB and LTBI were of interest given the region’s diversity of inhabitants, lack of urbanization, and increasing exposure to tourism. The age range for enrolment was 12 to 70 (mean = 38.5). Females accounted for 46% of the enrolled subjects. Individuals diagnosed with HIV/AIDS were excluded from this study. Previously, we reported on the lineage diversity of MTBC strains for TB-positive subjects, the low prevalence of *Mtb* isolates resistant to first-line antibiotic drugs, and high prevalence of LTBI among individuals without disease symptoms (Teklu et al., 2018a; Wondale et al., 2018; Teklu et al., 2018b). Using AFB smear microscopy, 70 specimens were identified as positive for MTBC. Of these, 53 specimens resulted in positive mycobacterial cultures. Two individuals were included in the TB cohort based only on clinical symptoms consistent with TB. MTBC lineage genotyping analysis resulted in the highest prevalences for the Euro-American (EA) and East-African-Indian (EAI) lineages (67 and 22%, respectively) (Wondale et al., 2020). The prevalence of LTBI was determined for 497 individuals enrolled in this study, frequently household contacts for subjects diagnosed with TB. The IGRA data suggested LTBI to be a frequent occurrence in this population (50.5% of all tested subjects). Based on the availability of simultaneously collected sputum samples sufficient for one or two types of analyses (proteomic and microbial) and the preference to have good matches to TB patients regarding gender and ethnicity, we conducted analyses here using samples from 100 individuals without evidence of TB. In total, 115 and 161 sputum samples were subjected to proteomic and microbiome surveys, respectively, as depicted in **Figure 1**. Individual specimen and human subject data categorized into the groups PTB, LTBI, and NCC are provided in **Supplementary File S1**.

### Sputum Proteomics

**Figure 2:**
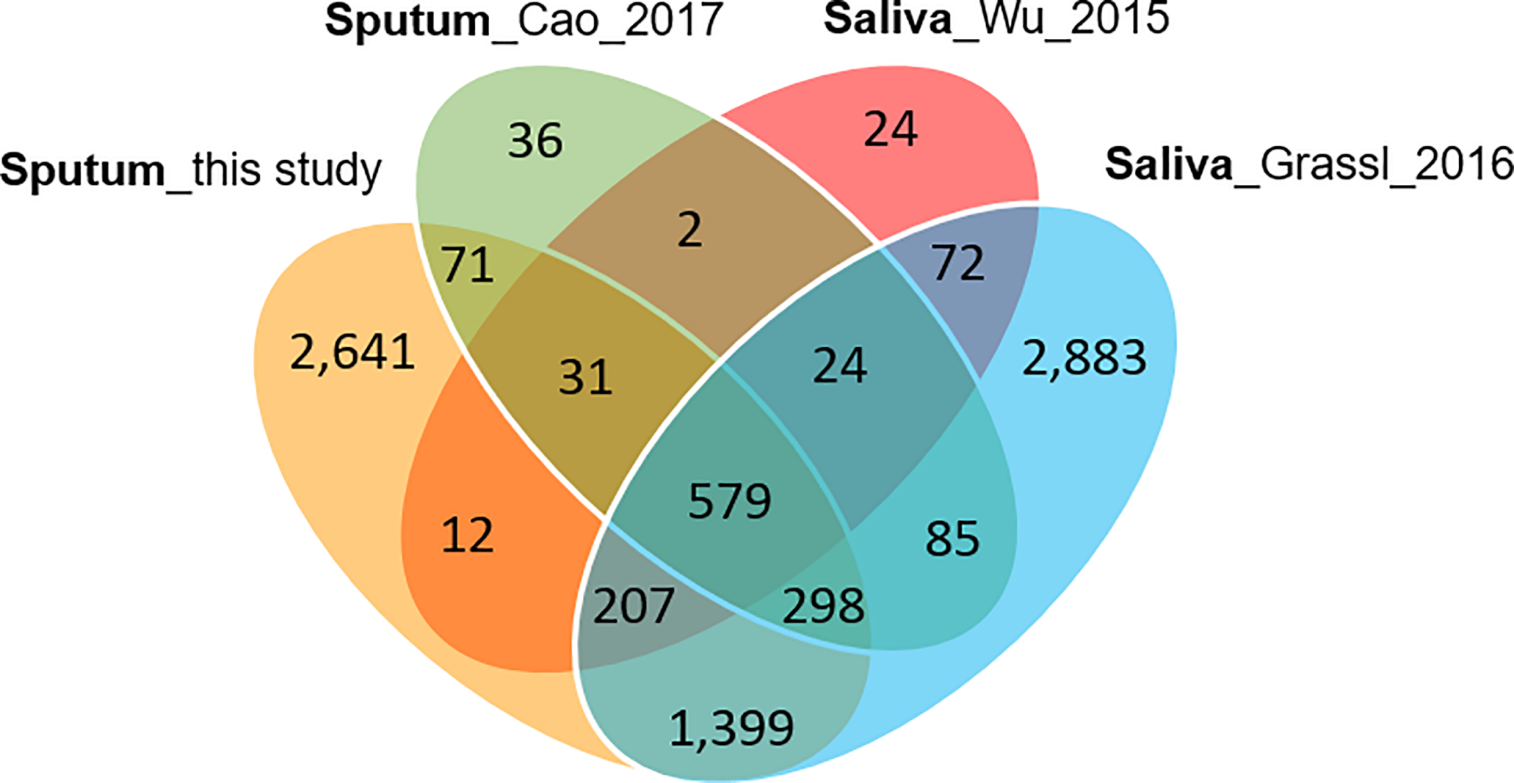
Venn diagram with protein identifications derived from two studies of saliva proteomes and two studies of sputum proteomes. The Cao study included analysis of samples associated with asthma. The Wu study included samples associated with squamous epithelial cell carcinoma of the oral cavity. In all studies, at least 800 proteins were identified.

Collapsing protein identifications (IDs) regardless of disease group, 2,039 and 207 non-redundant human and *Mtb* proteins, respectively, were obtained from the LC-MS/MS data. The numbers pertain to proteins with at least two unique peptides (**Supplementary File S2**). We note that mycobacterial proteins are not discussed in this report. LC-MS/MS technical replicates had higher correlation values for protein abundances than datasets comparing different biological samples (R-values of 0.93–0.98 *vs* 0.57–0.76, as shown in **Supplementary File S3**). This was indicative of good quantitative accuracy achieved by the proteomic workflow including computational analysis. To assess the protein contributions from upper respiratory tract (saliva) and lower respiratory tract (expectorated sputum), we compared our data with three other studies, two of those for saliva (Wu et al., 2015; Grassl et al., 2016; Cao et al., 2017). The Venn diagram in **Figure 2** displays protein ID overlaps among all datasets. As expected, protein ID overlaps of our study and the one by Cao et al. on a sputum proteome (Cao et al., 2017) were the highest (~87%). Sputum proteomes are composed of proteins originating from both upper and lower respiratory tracts. Examples of proteins that have been reported to be enriched in saliva and sputum, and are present in our surveys, are the basic salivary proline-rich protein 3 (PRB3) (Kim et al., 1993) and the pulmonary surfactant-associated protein A1 (SFTPA1) (Hermans and Bernard, 1999), respectively.

### Sputum Proteomics Reveals Neutrophil Infiltration and Acute Phase Responses in the Respiratory Tract of PTB Subjects

**Figure 3:**
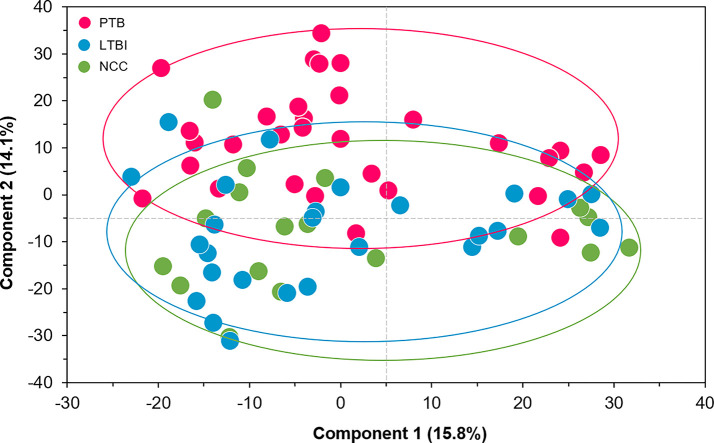
Principal Component Analysis (PCA) of sputum proteomes for PTB (red), LTBI (blue), and negative community controls (NCC, green). Two Principal Components explaining 15.8 and 14.1% of the variance among the groups are displayed. The data represent quantities of 432 human proteins. No separation of clusters is observed for the LTBI and NCC subjects while PTB datasets cluster separately.

**Figure 4:**
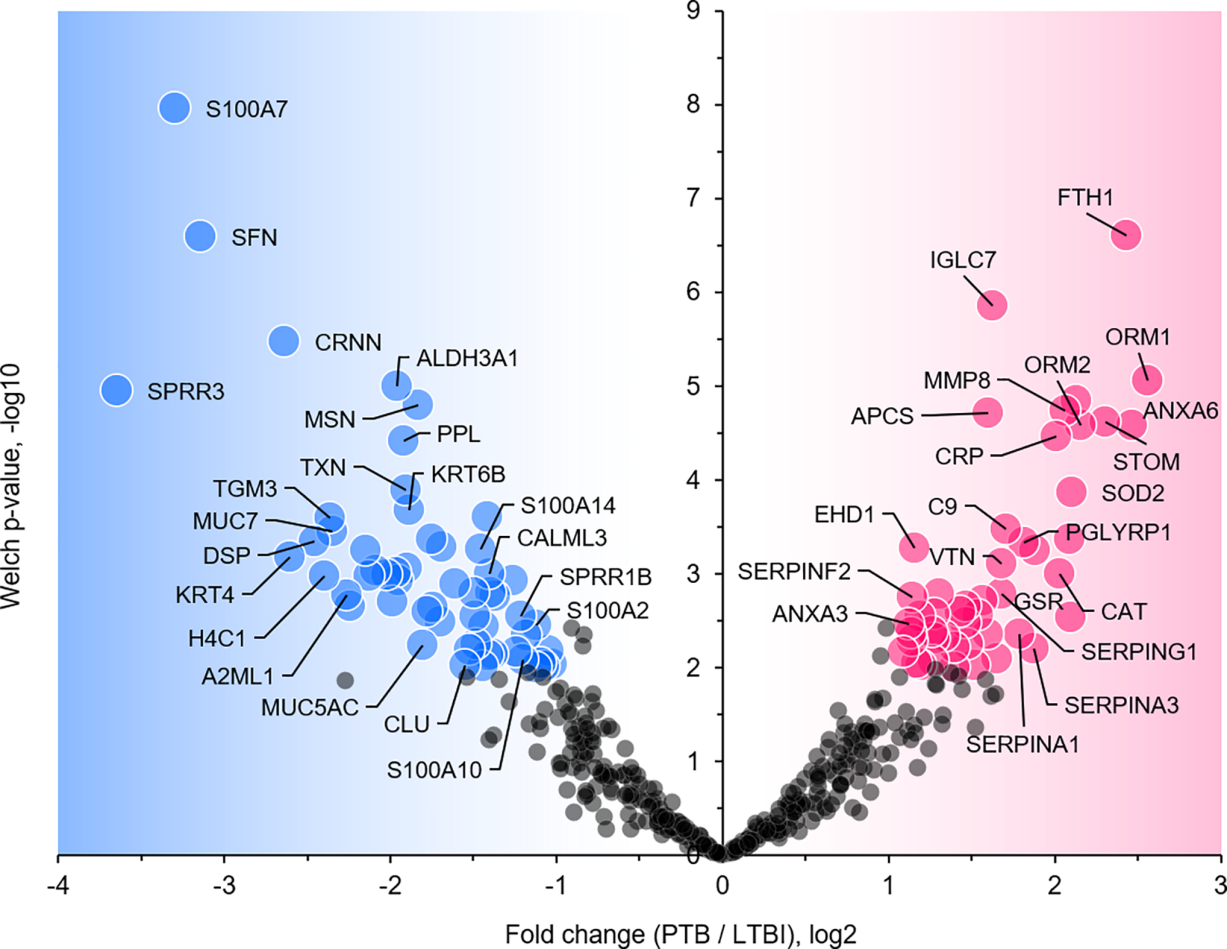
Volcano plot depicting protein abundance differences for the comparison of PTB and LTBI groups. Data are derived from sputum shotgun proteomic analyses. The unequal variance Welch t-test with multiple testing corrections was used in the Perseus software, and 103 proteins (marked with UniProt short names) had differences with a P-value <0.01 and a fold change >2. Red and blue dots denote proteins increased and decreased in the PTB group, respectively.

**Figure 5:**
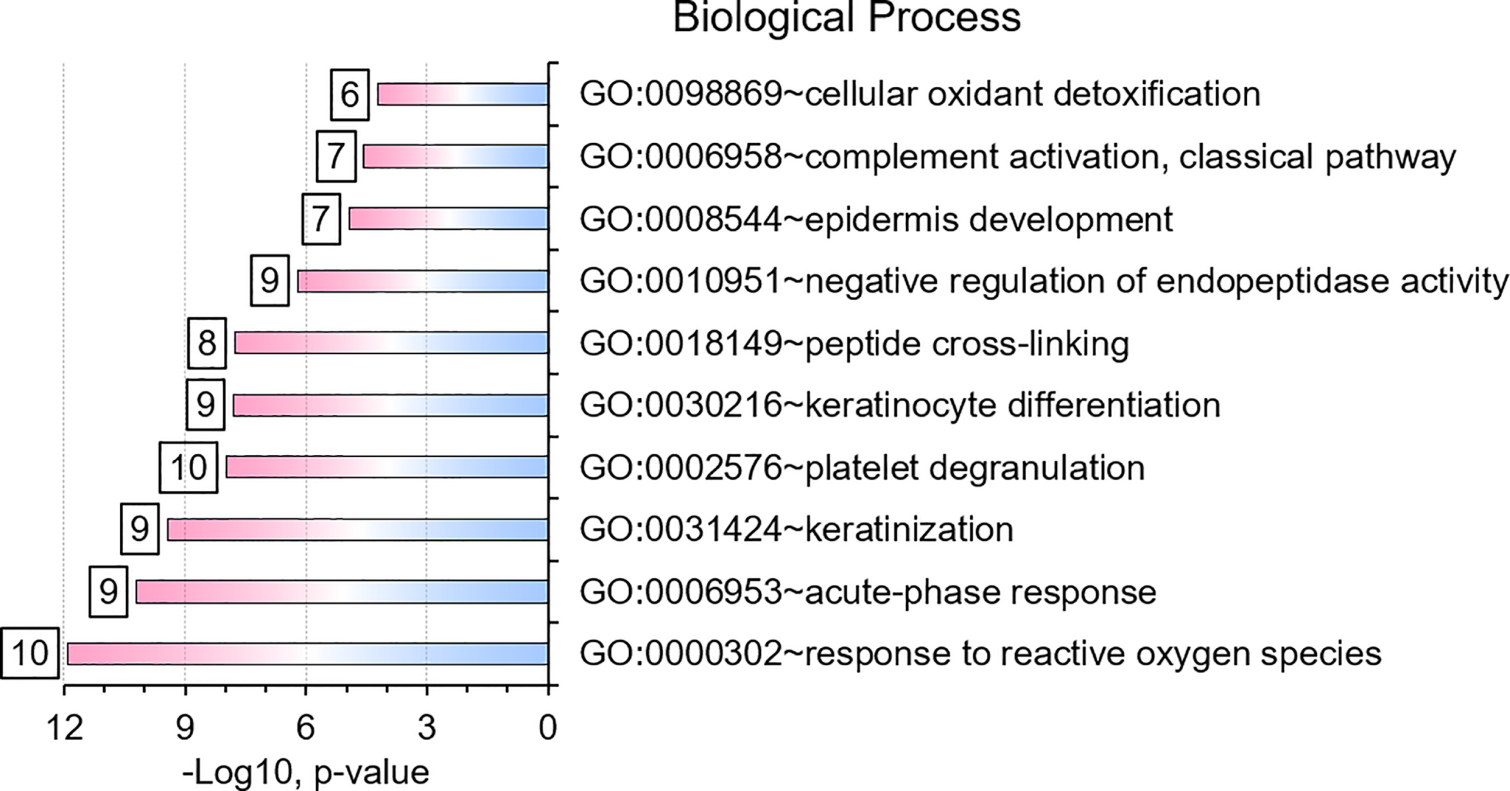
Gene Ontology (GO) biological process enrichments based on differentially abundant proteins (PTB *vs.* LTBI). The top-10 enriched terms and their significances (P-values) are plotted. The numbers placed next to a bar indicate the proteins in our datasets belonging to that category. GO term analysis details are included in a worksheet of a Supplemental Dataset (**Supplementary File S5**).

**Figure 6:**
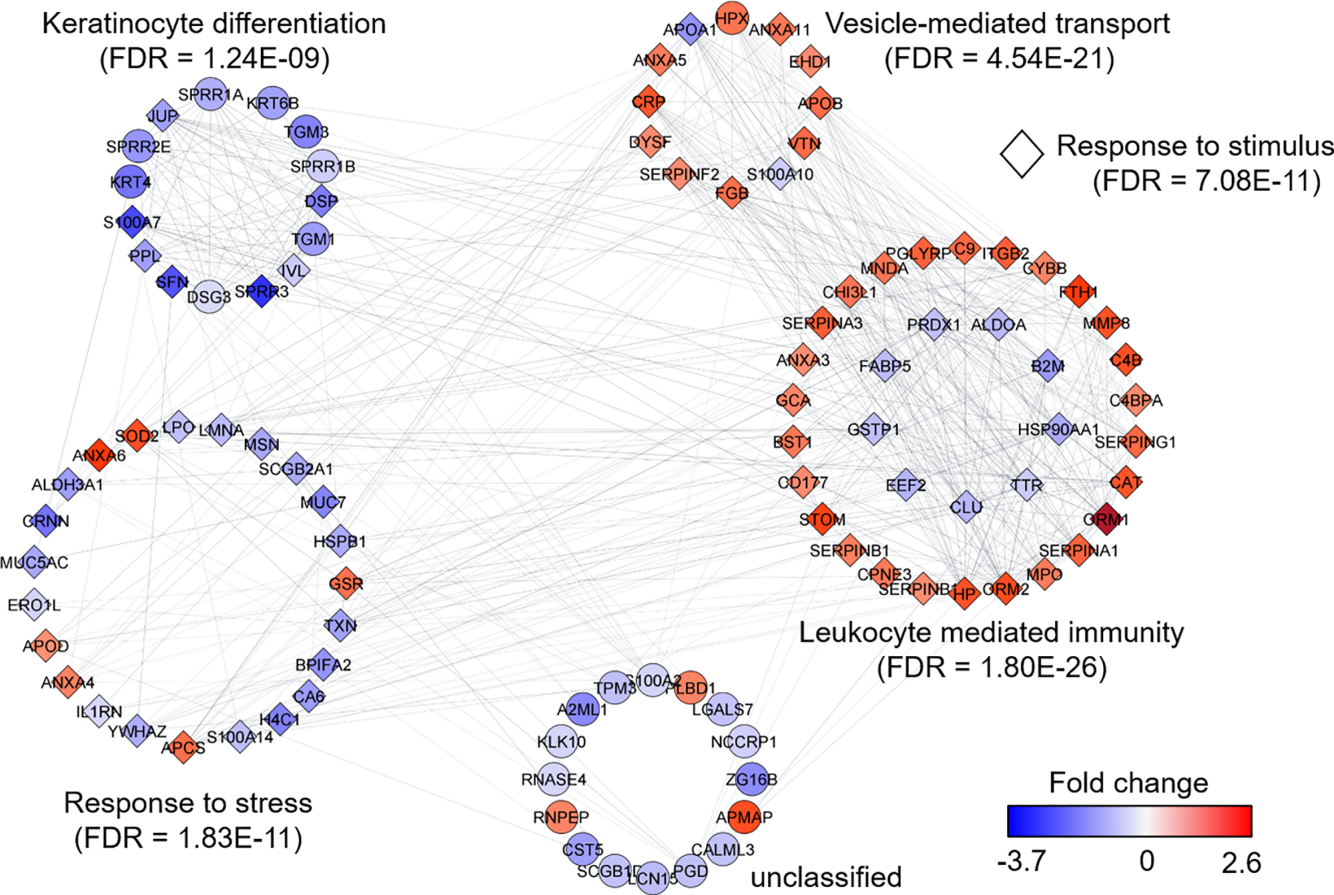
Protein network analysis and functional enrichment clusters. The network was built from 103 differentially abundant proteins comparing PTB and LTBI sample groups as input data and the String App in Cytoscape software. The score cut-off for interaction confidence was set to 0.4. Color coding is in accordance with the fold changes. Diamond shape depicts proteins associated with a response to stimulus. Protein clusters were annotated based on enrichment, a function embedded in Cytoscape.

We selected datasets with at least 150 protein IDs per sample and protein IDs detected in at least nine subjects. Thus, 75 datasets were retained for quantification and clustering analyses, each with 432 distinct proteins (**Supplementary File S2**). Pearson Correlation hierarchical clustering (HCPC) was used to assess if gender-specific omics analyses were warranted. While HCPC did not show significant gender-specific clustering, clusters were observed based on the diagnosis of infection with PTB (**Supplementary File S4**). Next, gender-integrated data were submitted to Principal Component Analysis (PCA). PTB datasets largely clustered separately, although some overlap with the LTBI and NCC groups was observed (**Figure 3**). Lack of separation between LTBI and NCC datasets in the PCA suggested that latency of TB does not strongly influence the sputum proteome compared to this disease’s absence. Differential sputum proteomic analysis using unequal variance Welsh t-tests (Welch, 1947) resulted in 103 proteins with ≥2-fold changes (with Benjamini-Hochberg method corrected P-values <0.01; PTB *vs* LTBI data). Twelve of these proteins are listed in **Table 1**. All 103 proteins are displayed in the Volcano Plot of **Figure 4** and listed in the **Supplementary File S5**. Examples of antimicrobial effectors increased in TB datasets are peptidoglycan recognition protein 1, collagenase MMP8, and myeloperoxidase (MPO). Detailed data on protein quantification, tissue localizations, and functional roles are presented in **Supplementary File S6**. This data supported strong inflammatory and immune responses against *Mtb* for the PTB cohort only: 16 and 14 of 47 proteins that were quantitatively increased in the PTB group (*vs* LTBI) are acute phase reactants or highly expressed in leukocytes, respectively. Protein Atlas tissue expression profiles supported the notion that these leukocyte-associated proteins were derived from neutrophils. Four proteins revealed consensus data in Protein Atlas (Uhlen et al., 2015) for unique presence in neutrophil granules or membranes (see **Supplementary File S6**). Other differentially abundant proteins contribute to oxidative stress responses (e.g., superoxide dismutase 2, apolipoprotein D, mitochondrial glutathione reductase and catalase). The GO term biological process enrichment (**Figure 5**) and protein network analysis in Cytoscape (**Figure 6**) were consistent with leukocyte-, primarily neutrophil-mediated immune activation and acute phase response pathways. Neutrophils are responsible for inflammation and kill pathogens using various mechanisms while acute phase and oxidative stress responses limit host cell collateral damage. Of the 56 proteins decreased in the PTB datasets in comparison to the LTBI datasets, half are expressed, and often enriched, in esophageal and oral mucosal tissues (**Table 1** and **Supplementary File S6**). Four proteins are specifically secreted by salivary glands. Respiratory mucosal cells express and release proteins dedicated to form a barrier towards the external environment. Protein network analysis and enriched GO term categories yielded evidence that relative quantitative increases of the 56 proteins (LTBI *vs* PTB) are associated with processes reflecting the normal physiology of the respiratory tract squamous epithelium such as keratinocyte differentiation, peptide cross-linking, stress responses, and epidermal development (**Figures 5** and **6**). Given that inflammatory cells and their proteins are released into sputum during anti-*Mtb* responses, those that represent the normal squamous epithelial secretion and shedding of dead cells are reduced in abundance, relatively to the LTBI group. Examples of the proteins that represent the aforementioned processes are psoriasin (protein S100-A7), two mucins, two small proline-rich proteins, and additional cytoskeleton-associated cornified envelope proteins such as periplakin, desmoplakin, cornulin, and plakoglobin (**Supplementary Files S5**, **S6**). These findings are likely not biologically important since they reflect contamination of saliva in the sputum samples.

**Table 1:** Subset of differentially abundant proteins (PTB ***vs*** LTBI).

Protein name^1^	Protein SN^2^	Fold-change	Welch test^3^, −log10 *P*-value	Extracellular or cellular functions^4^	Body fluid and tissue enrichment^5^
Ferritin	FTH1	5.39	6.60	Acute phase response	NA
α-1-Acid glycoprotein 1	ORM1	5.89	5.06	Acute phase response	Plasma, *alveolar macrophages (Fournier et al., 1999)
α-1-Acid glycoprotein 2	ORM2	4.46	4.59	Acute phase response	Plasma, *alveolar macrophages (Fournier et al., 1999)
Neutrophil collagenase	MMP8	4.19	4.74	ECM degradation	*Neutrophils (Hasty et al., 1990)
Peptidoglycan recognition protein 1	PGLYRP1	3.52	3.34	Bactericidal protein recognizing cell wall	*Neutrophils (Read et al., 2015)
C-reactive protein	CRP	4.03	4.47	Acute phase response	Plasma (Gould and Weiser, 2001)
Protein S100-A7	S100A7	0.10	7.96	Antimicrobial, Keratinization	Esophagus, oral mucosa, tonsils
14-3-3 protein sigma	SFN	0.11	6.59	Keratinization, cell proliferation	Esophagus, oral mucosa, tonsils
Cornulin	CRNN	0.16	5.48	Keratinization, Cell proliferation	Esophagus, oral mucosa, tonsils
Aldehyde dehydrogenase 3A	ALDH3A1	0.26	4.99	Detoxification, Cell proliferation	Esophagus, oral mucosa, tonsils
Mucin-7	MUC7	0.20	3.45	Cell-protective in upper respiratory tract cells	Salivary glands (Xu et al., 2016)
Small proline-rich protein 3 (esophagin)	SPRR3	0.08	4.95	Keratinization	Esophagus, oral mucosa, tonsils

Twelve proteins with high statistical significance comparing abundances in PTB vs LTBI proteome datasets and a fold change of >3.5 (Welch test). ^1^common protein name; ^2^short name as listed in UniProt database; ^3^negative logarithmic P-value; ^4^functions derived from UniProt database entries or literature; ^5^enrichment in human body fluid, cell type, or tissue [if no reference listed, data from Protein Atlas database (Uhlen et al., 2015)].

### α-1-Acid Glycoproteins

**Figure 7:**
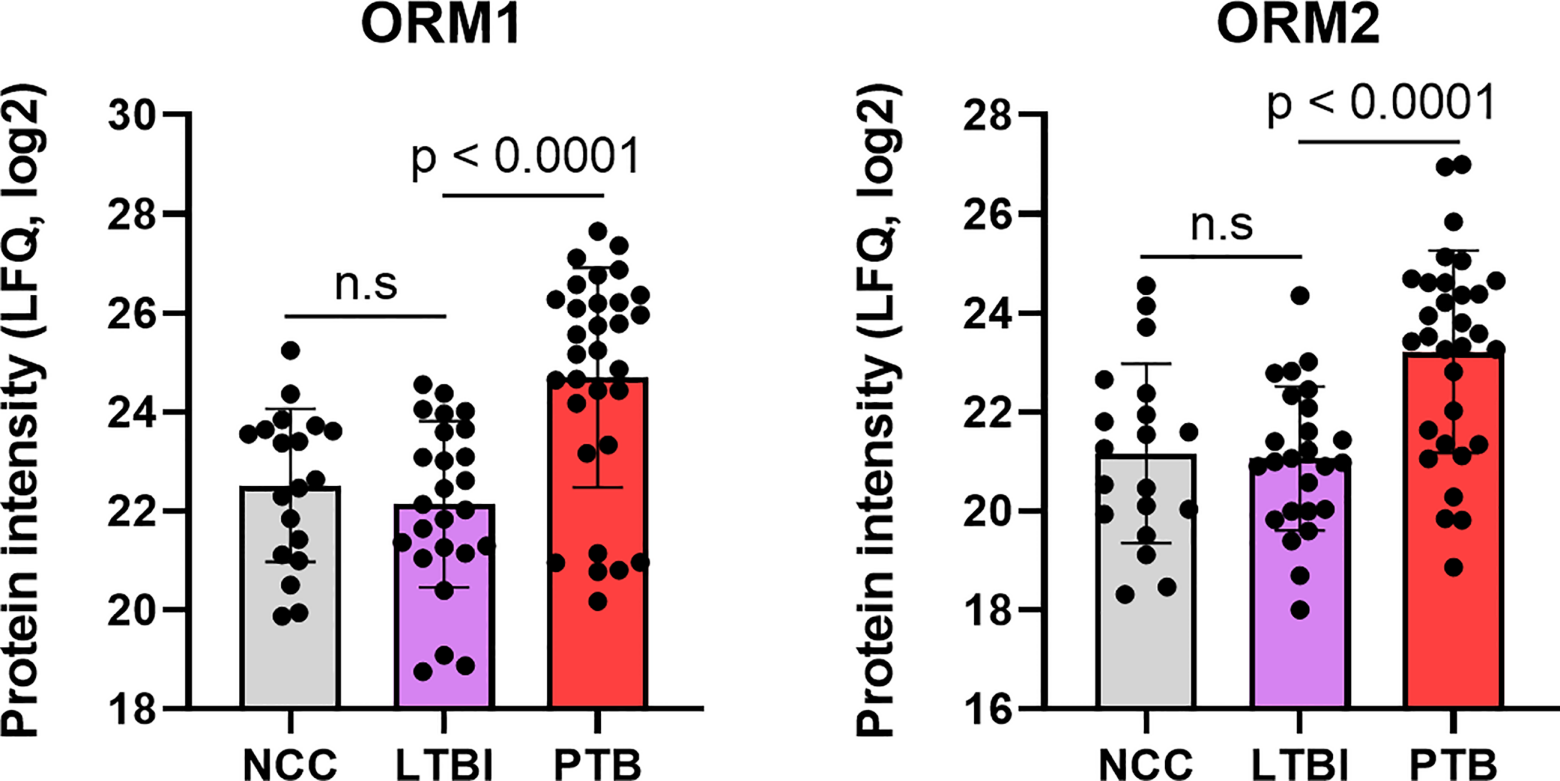
Quantitative differences for the α-1-acid glycoproteins ORM1 and ORM2 in box plots comparing datasets for PTB *vs* LTBI as well as PTB *vs* NCC. The P-values were highly significant indicating the important role for the acute phase reactants in modulating the PTB pathology. LFQ values are based on summed MS1 peak integrations for all peptides assigned to protein of origin. n.s, not statistically significant.

Complement system activation and negative regulation of endopeptidase activities are elements of the acute phase response. Proteins with these functions were increased in the PTB group, e.g., complement factors C4B and C9, and the serpins A1, A3, B1, B10, F2, and G1 (**Figure 4**) and enriched as a GO term (**Figure 5**). In addition to ferritin, two isoforms of α-1-acid glycoprotein (α1-AGP), also named ORM1 and ORM2, had greater than 4.4-fold increases in the PTB group (**Figures 4**, **7**) while no such differences were evident in the comparison of LTBI and NCC groups. α1-AGP is released by alveolar macrophages during pulmonary inflammation (Fournier et al., 1999), in addition to its secretion by hepatocytes into blood plasma. Western Blots for 18 sputum samples revealed a wide range of M_r_ values for α1-AGP and made proteomic data validation in a defined M_r_ range difficult (**Supplementary File S7**). But the α1-AGP bands in PTB samples had overall higher staining intensities than LTBI and NCC samples.

### Sputum Proteomic Analyses Reveal a Few Abundance Differences Comparing LTBI and NCC Groups

**Figure 8:**
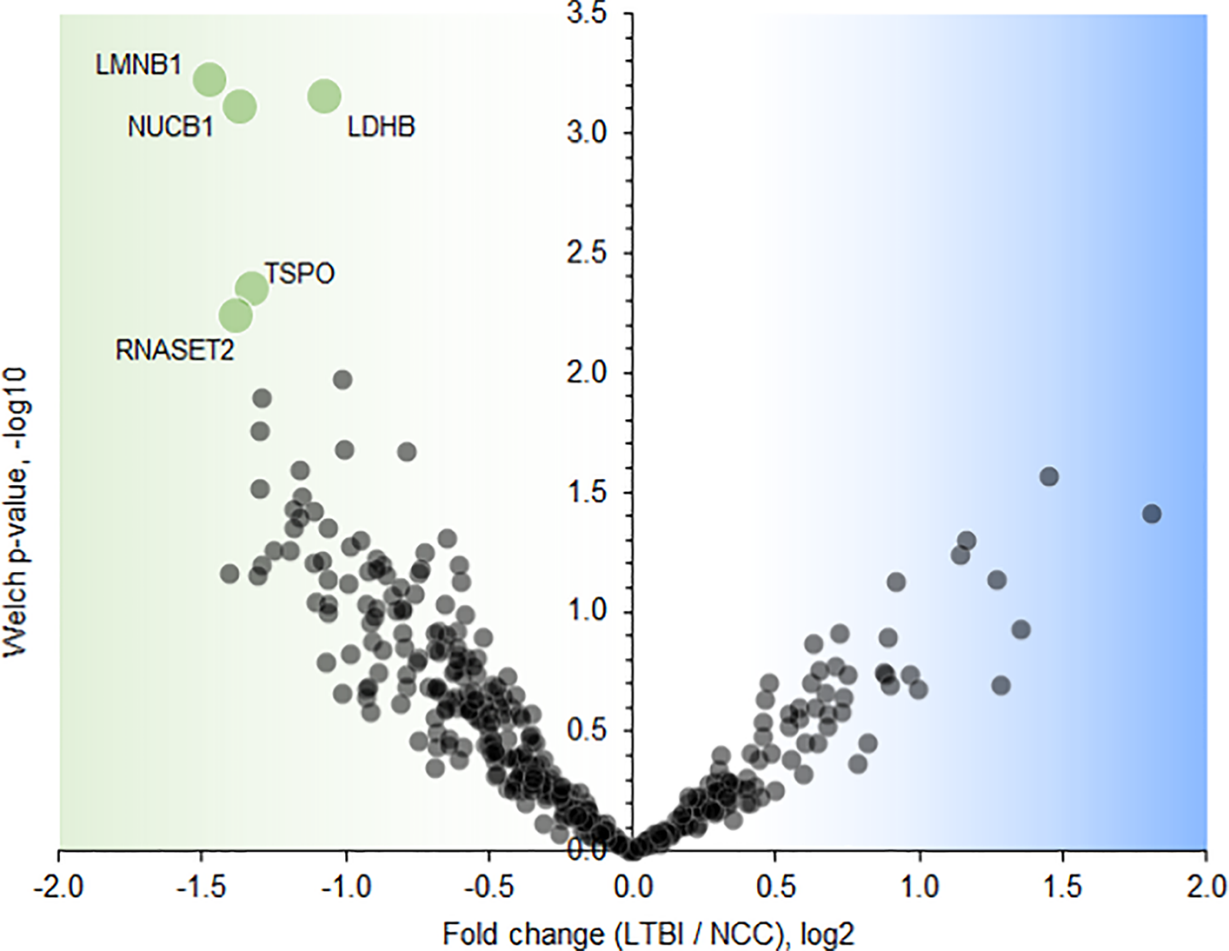
Volcano plot depicting protein abundance differences comparing the LTBI and NCC groups. A P-value <0.01 and a fold change >2 were applied to identify the differentially abundant proteins (each denoted in green and marked with the UniProt short name).

Twenty-one proteins were differentially abundant comparing IGRA-positive (LTBI) and NCC datasets (**Figure 8**). Lamin B1 (LMNB1), nucleobindin-1 (NUCB1), ribonuclease T2 (RNASET2), lactate dehydrogenase B chain (LDHB), and translocator protein (TSPO) displayed the highest statistical significances. Small proline-rich protein 3 (SPRR3) was the only protein that differed in abundance as it pertains to the TB *vs* LTBI and the LTBI *vs* NCC comparisons (**Table 2**). It is noteworthy that two of the aforementioned proteins are enriched in macrophages and monocytes according to the Protein Atlas entries (TSPO and RNASET2). An integrated functional context of this set of proteins was not apparent. Detailed data on differentially abundant proteins are provided in **Supplementary File S6** (all protein characteristics and their abundance ratios) and **Supplementary File S8** (proteins depicted in **Figure 8**). They are of interest as surrogate biomarkers in sputum to discern the latent TB disease stage from healthy human subjects. Given that sample groupings were derived from IFN-γ release data, inaccuracies associated with those measurements would adversely affect the differential analysis of sputum proteomic data (LTBI *vs* NCC).

**Table 2:** Differentially abundant proteins (LTBI *vs* NCC).

Protein name^1^	Protein SN^2^	Fold-change	Welch test^3^, −log10 *P*-value	Extracellular or cellular functions^4^	Body fluid and tissue enrichment^5^
Lamin B1	LMNB1	0.36	3.23	Nuclear envelope breakdown	NA
Ribonuclease T2	RNASET2	0.38	2.24	Recognizes and degrades microbial pathogen RNAs	Plasma, monocytes
Nucleobindin-1	NUCB1	0.39	3.11	Calcium-binding and signal transduction	Plasma
Translocator protein	TSPO	0.40	2.35	Transport of porphyrins, heme, and cholesterol	Blood, macrophages and monocytes
L-Lactate dehydrogenase B chain	LDHB	0.47	3.15	Anaerobic metabolic pathway	NA
Small proline-rich protein 3 (esophagin)*	SPRR3	3.50	1.41	Keratinization	Esophagus, oral mucosa, tonsils

Six proteins with high statistical significance (Welch test), comparing abundances in LTBI vs NCC proteome datasets. *moderate statistical significance but of interest due to change in PTB vs LTBI and LTBI vs NCC. ^1^common protein name; ^2^short name as listed in UniProt database; ^3^negative logarithmic P-value; ^4^functions derived from UniProt database entries or literature; ^5^enrichment in human body fluid, cell type, or tissue [data from Protein Atlas database (Uhlen et al., 2015)].

### Microbiome Comparisons Reveal Differential Abundances of *Rothia* and *Haemophilus* for PTB and LTBI Cohorts

**Figure 9:**
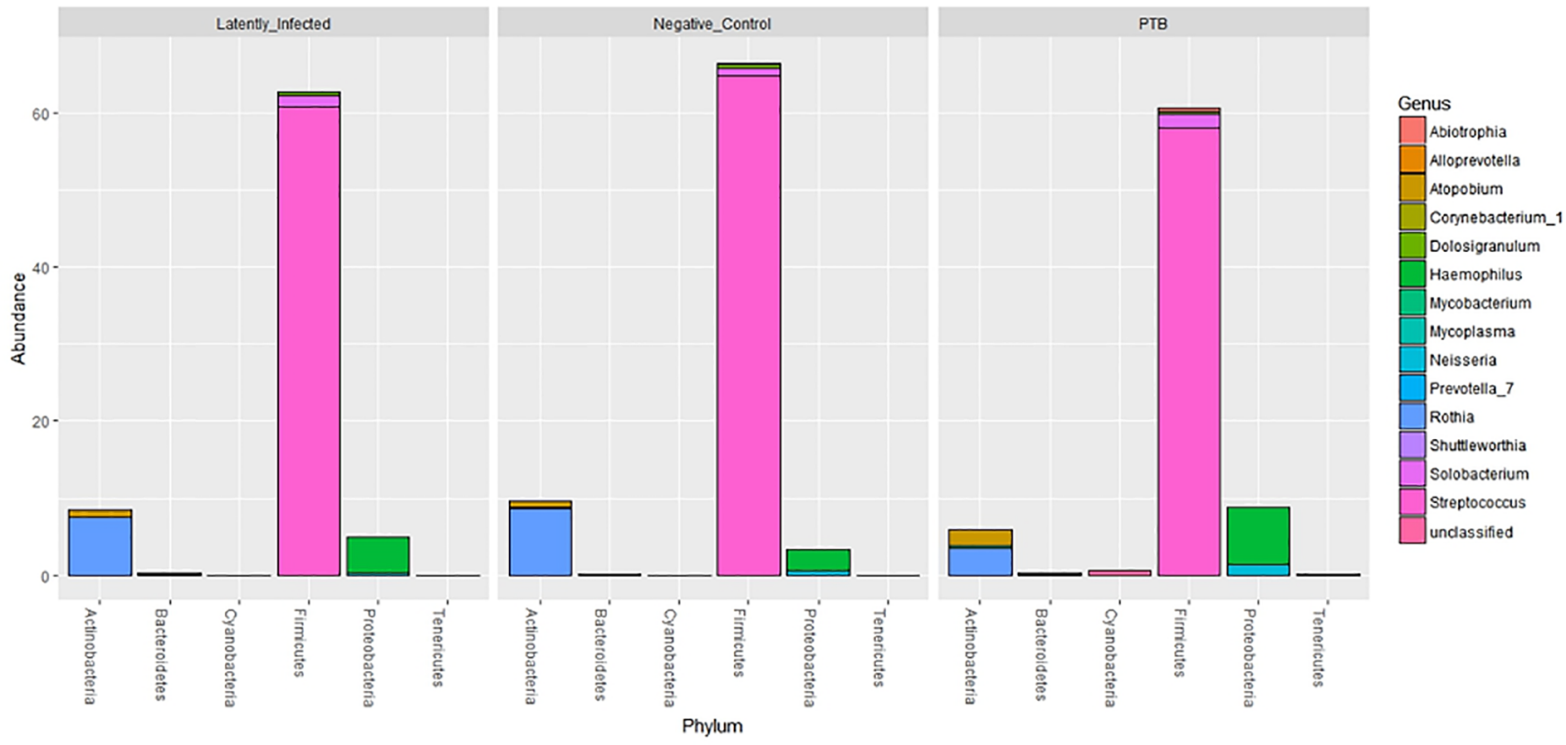
Microbial taxonomy profiling performed at the phylum level denoting the differentially abundant genera based on sequence analysis of the V4 region of 16S bacterial rRNA on a MiSeq platform. The phyla are shown denoting the most abundant genera for each phylum by color codes. *Streptococcus* is the most abundant genus of Firmicutes and dominant in oral microbiota. Among Actinobacteria, *Rothia* and *Atopobium* were dominant with variations in abundance among LTBI, NCC, and PTB datasets. *Mycobacterium*, also an Actinobacterium, revealed low abundance so that it is not visualized in the segmented bars for this phylum. *Haemophilus* was the most abundant genus, followed by *Neisseria*, in the phylum Proteobacteria.

**Figure 10:**
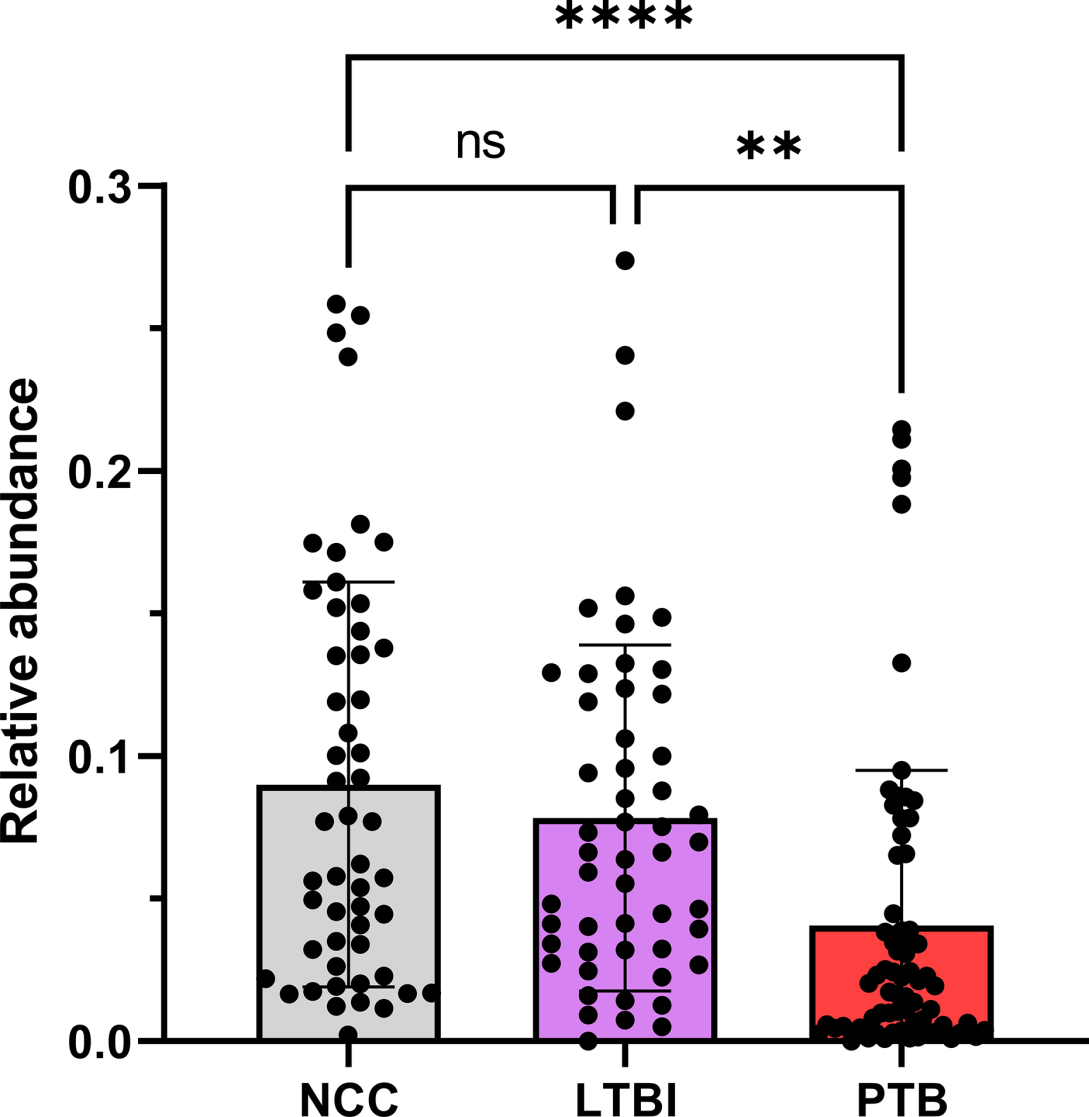
Quantitative differences for *Rothia* displayed in box plots comparing PTB datasets with those of the LTBI and NCC groups. As shown, the *P*-values were significant in both comparisons. ****P-value < 0.0001; **P-value < 0.01; ns, not statistically significant.

Using the V4 region of 16S rRNA to classify sputum microbial taxa, we determined *Streptococcus* to be the most abundant genus (**Figure 9**) and noted the absence of α-diversity differences at the level of genera (**Supplementary File S9**) among the cohorts. There was no separation of the oral microbial profiles among the three cohorts based on the PCA data (**Supplementary File S10**). *Mycobacterium*, as a genus, was detected only for individuals in the PTB group (adjusted P-value of 3 × 10^−11^, PTB *vs* LTBI). This finding generated confidence in the accuracy of the V4-region 16S rRNA sequence analysis (**Supplementary File S11**). We identified significant quantitative differences for the taxa *Rothia* and *Haemophilus*. The genus *Rothia* featured a three-fold decrease with an adjusted *P*-value of 8.9 × 10^−8^ (PTB *vs* LTBI, **Figure 10**). The genus *Haemophilus* had a 2.2-fold increase with an adjusted *P*-value of 0.029 (PTB *vs* LTBI). Interestingly, both *Rothia* and *Haemophilus* were also the on average most abundant genera for their respective phyla, Actinobacteria and Proteobacteria (**Figure 9**). We identified 13 additional genera with statistically significant differences (adjusted *P*-values <0.05), but the fold changes were either lower than 1.5 or the genera had low sequence read assignments (**Supplementary File S12**).

## Discussion

### Neutrophil Effectors and APR Proteins Are Sputum Biomarkers for PTB

We report quantitative differences for proteins and microbial taxa derived from sputum samples comparing a PTB cohort from South Omo with asymptomatic cohorts, one of which was designated as LTBI based on our data from a WHO-approved IGRA (Bastian et al., 2017). The study followed investigations of lineage analysis of MTBC isolates derived from individuals with TB as well as cohort and immunological characterizations of the LTBI group from this multi-ethnic pastoralist region of Ethiopia (Wondale et al., 2017; Teklu et al., 2018a; Wondale et al., 2018; Teklu et al., 2018b). The PCA revealed good separation of PTB proteome profiles from those of the other groups. LTBI and NCC clusters were not discerned. More than 100 differentially abundant proteins (PTB vs LTBI) allowed insights into immune responses linked to the PTB infections (**Figures 4**–**6**). Functional and network analysis of these proteins is highly consistent with the infiltration of neutrophils, and perhaps other leukocytes in the respiratory tract and the release of antimicrobial effectors into sputum for subjects with PTB. Examples of such effectors are peptidoglycan recognition protein 1, the collagenase MMP8, and MPO. Proteins part of the complement system (e.g., C4B, C9) and the APR were also increased in abundance in the PTB cohort. Furthermore, innate immune response pathways were apparently activated to eliminate the pathogen and limit or resolve host cell damage. To counteract the inflammation-generating effects of immune effectors, protease inhibitors were elevated in abundance in the PTB compared to the LTBI group; this includes serpin B1, which inhibits neutrophil proteases and modulates innate immunity (Benarafa et al., 2007; Benarafa et al., 2011), serpin B10, which appears to control TNF-α-induced cell death (Schleef and Chuang, 2000); and serpin G1, which inhibits complement activity and other pro-inflammatory signals (Dorresteijn et al., 2010). Oxidative stress response enzymes including catalase, SOD2, and ApoD were also increased in the PTB group, suggesting a role in controlling damage by ROS. ROS levels rise when leukocytes degranulate and activate NADPH oxidase (subunit CYBB was increased in abundance in the PTB group) and MPO. High density lipoproteins harboring ApoD and ApoB (also increased in abundance in the PTB group) are known to suppress TNF-α release from *Mtb*-infected macrophages (Inoue et al., 2018). Two APR proteins that scavenge heme and iron extracellularly (hemopexin and ferritin, respectively) were elevated in abundance in sputum of subjects with PTB compared to LTBI. Sequestering iron/heme limits the growth of pathogens in infected tissues (Parrow et al., 2013). *Mtb* is able to enter a persistent state in necrotic granulomas under iron sequestration challenges (Kurthkoti et al., 2017), thus enabling long-term survival in the host. Given that sputum proteome profiles of the LTBI group were much more similar to the NCC group than to the PTB group, the host defense and inflammation-associated pathways were apparently absent, or immeasurably small, during latent infection. The systemic role of neutrophils in the response to PTB was linked to a neutrophil-driven IFN-γ inducible transcriptional signature in whole blood that correlated with disease severity (Berry et al., 2010). The important role of neutrophils as phagocytes critical to the defense against lung-invading *Mtb* was previously reported (Eum et al., 2010). Whether persistent neutrophil activation is detrimental to the clinical outcomes of chronic TB manifestations is a matter of debate (Lowe et al., 2012). Since TB severity was not assessed in our work, we were unable to correlate the APR and neutrophil biomarkers with the severity of infection.

### Proteins of Epithelial and Salivary Gland Origin Were Lower in Abundance in PTB *vs* LTBI Subjects

Since label-free LC-MS/MS quantification calculates protein contributions relative to total proteome, we argue that decreased abundances of these proteins in sputum of PTB patients is a consequence of infiltration, degranulation, and lysis of immune cells along with APR proteins that are secreted *via* the microvasculature into the airways. It was reported that viral respiratory infections induce mucin secretion to enable trapping the viruses in mucus (Vareille et al., 2011). We do not see an analogy to *Mtb* infection. Two mucins secreted into the respiratory tract (MUC7 and MUC5AC) were actually less abundant in PTB compared to LTBI sputum profiles. Psoriasin (S100-A7), a squamous epithelial protein also decreased in PTB datasets has antimicrobial and neutrophil-degranulating functions in the upper airways (Glaser et al., 2009). Our data do not support a role of S100-A7 in the anti-*Mtb* response. We surmise that *Mtb* infection does not trigger host defenses in the mucosa of the upper airways, which is in line with the finding that upper respiratory tract TB is a rare clinical event (Jindal et al., 2016).

### α-1-Acid Glycoproteins Are Strong Sputum Biomarkers for PTB

APR proteins were invariably higher in abundance in sputum of PTB compared to LTBI subjects. APR protein increases were previously linked to *Mtb* infections and a biomarker role: serum α-1-antitrypsin in the context of TB diagnosis (Song et al., 2014); serum CRP and ferritin as biomarkers of disease persistence during anti-TB therapy (Miranda et al., 2017). Ferritin concentrations were reported to be increased in acute inflammatory lung injury in order to bind extracellular and intracellular iron to reduce host susceptibility to oxidative damage (Kim and Wessling-Resnick, 2012). Ferritin was also associated with leakage from damaged cells (Kell and Pretorius, 2014). In addition to ferritin, we found two α1-AGP proteins (ORM1 and ORM2) to be elevated in abundance in PTB patients. Earlier, α1-AGP was described as induced in alveolar macrophages upon lung inflammation, with a 4-fold increased secretion in patients afflicted by interstitial lung injuries (Fournier et al., 1999). A murine study identified foamy macrophages located in pneumonic areas of *Mtb*-infected lungs as an important source of α1-AGP; administration of antibodies neutralizing this glycoprotein led to lower bacillary loads and less tissue damage, suggesting an adverse effect of α1-AGP during disease progression (Martinez Cordero et al., 2008). We surmise that sputum α1-AGPs and ferritin are surrogate biomarkers of PTB and also influence the outcome of *Mtb* infections. The contribution of α1-AGPs to the immunopathology of PTB needs to be evaluated further in disease models. Their roles as sensitive and specific protein biomarkers in sputum, in the context of early diagnosis or disease progression, requires further validation by surveying larger cohorts.

### LTBI Biomarker Candidates

Five proteins were differentially abundant comparing LTBI and NCC cohorts with low *P*-values (<0.01), all of them less abundant in the sputum of the LTBI group. These proteins are candidate biomarkers for the diagnosis of LTBI, perhaps complementary to IGRAs. The specificity and negative and positive predictive values of IGRAs were reported to be high for LTBI diagnosis (Diel et al., 2011). However, the test kits are expensive and moderate performance was noted for their sensitivity to diagnose latent disease in children in the 55–70% range (Machingaidze et al., 2011). With respect to the proteins identified by our differential proteomic analysis, only the translocator protein TSPO was previously linked to the pathology and a biomarker role (monitoring TB progression *in situ*) (Foss et al., 2013). Radioiodinated DPA-713, a TSPO synthetic ligand, was used to visualize anti-TB host responses *in vivo*. Strong TSPO and CD68 co-staining was observed for macrophages in granulomas (Foss et al., 2013). TSPO, a protein enriched in mitochondrial outer membranes (Betlazar et al., 2020), is also endogenously expressed in bronchoalveolar epithelial cells (Hardwick et al., 2005), mediates cholesterol translocation, and was physiologically linked to neuroinflammation by influencing MAPK pathways and the NLRP3 inflammasome (Betlazar et al., 2020). Interestingly, *Mtb* activates the NLRP3 inflammasome *via* the ESX‐1 secretion system (Dorhoi et al., 2012). TSPO was not differentially abundant in our PTB *vs* LTBI cohort comparison. We hypothesize that low abundance of TSPO in macrophages alters the crosstalk of pathogen and immune cells in infected lungs, but this does not explain why there would be quantitative differences for this protein between LTBI and NCC cohorts. LDHB, another protein decreased in the LTBI *vs* NCC cohort, may also influence anti-*Mtb* immune responses. The mitochondrial enzyme’s product, lactate, was reported to promote the switch of CD4 T cells to an IL-17 T cell subset and reduce CD8 T cell cytolytic capacity (Pucino et al., 2017). A third protein was ribonuclease T2, part of a family of ribonucleases that have immunomodulatory and antimicrobial properties (Lu et al., 2018).

### *Rothia*, a Respiratory Tract Microbial Biomarker for PTB?

We conducted one of the two largest microbiome profiling efforts related to TB to date. While there were no differences in overall α- or β-diversity in sputum microbiomes among the cohorts, we identified statistically significant differences in abundance for the genera *Rothia* and *Haemophilus*, two oral microbiome community members, in the comparison of the PTB and LTBI datasets. *Rothia mucilaginosa* was recently reported to produce the siderophore enterobactin in the human oral niche (Uranga et al., 2020). *Mtb* is also a producer of siderophores (mycobactins), which are released to acquire iron from the host in infected lung tissue (De Voss et al., 2000). Enhanced iron sequestration during an active host inflammatory response in PTB possibly diminishes the fitness and growth of *Rothia* species in the upper respiratory tract. This genus is a ubiquitous commensal organism of the oral cavity (Singh et al., 2019; Uranga et al., 2020). One may also speculate that the genus *Haemophilus*, increased in abundance in respiratory secretions of PTB compared to LTBI subjects, is due to its better adaptation to inflammatory conditions caused by the infection with *Mtb*. The pathogen *Haemophilus influenzae* adapts to the neutrophil-rich milieu in the inflamed airways of asthmatic patients (Essilfie et al., 2012). Our 16S rRNA analysis did not allow further insights at the species level. Recently, a comprehensive multi-center study assessing sputum metagenomic profiles from TB patients in comparison with healthy controls and patients diagnosed with other lung diseases reported the absence of distinct, common microbial signatures for TB patients across three geographical locations (Italy, Switzerland, and Bangladesh) (Sala et al., 2020). Consistent with our findings, differences in microbial α-diversity comparing the TB and healthy control cohorts were not observed. We note that different regions of the 16S rRNA gene were profiled in our and the study by Sala et al. We sequenced the V4 region while Sala et al. (2020) sequenced the V1-V2 region. Other oral microbiome characterization studies comparing TB and control cohorts did not report statistically significant differences for the genera *Rothia* and *Haemophilus* (Naidoo et al., 2019). One study analyzing nearly 100 sputum specimens (Wu et al., 2013) associated respiratory microbiota with different treatment outcomes for TB. Subjects with LTBI were not included. The most significant genus-level change was *Prevotella*, an anaerobic bacterium with higher abundance in healthy controls compared to subjects with TB (Wu et al., 2013). A recent intriguing discovery was the role of *Helicobacter* colonization of the gut in IFN-γ-dependent reduced susceptibility to active TB progression, observed in a primate study that may translate to *Helicobacter* colonization in humans (Perry et al., 2010). The production of metabolites by anaerobic respiratory bacteria was discussed as a potential modulatory influence on risk of TB progression (Naidoo et al., 2019). Given the high compositional variability of oral and gut microbiota for different socio-economic and geographic settings, such comparisons remain challenging. Our study was plausibly influenced by the diets of the highly diverse participating ethnic groups and the occurrence of malnutrition. Thus, further studies will be needed to determine the functional relevance of *Rothia* and *Haemophilus* abundance changes in the context of PTB and inflammation of the human airways.

## Conclusions

We performed comprehensive analyses of sputum microbiomes and proteomes comparing Ethiopian cohorts characterized by high cultural and ethnic diversity in a remote region to learn more about molecular and microbial community differences related to acute and latent infections with *Mtb*. In the comparison of three groups (TB, LTBI, NCC) we discovered new and verified previously reported protein biomarkers that are useful as targets of sputum diagnostic tests for active TB. Additionally, we identified potential protein biomarkers for latent TB that are interesting targets to complement or even replace the currently WHO-recommended diagnostic tests for LTBI, IGRA assays. We identified a potentially antagonistic relationship between *Mtb* and *Rothia* based on strongly decreased, statistically significant *Rothia* abundance decreases in the TB *vs* LTBI and NCC cohorts. Our findings need to be validated in larger clinical studies and deserve additional mechanistic investigations.

## Data Availability Statement

The datasets presented in this study can be found in online repositories. The 16S rDNA sequencing data were deposited in the NCBI BioProject under dataset identifier PRJNA663902. URL: https://www.ncbi.nlm.nih.gov/bioproject/PRJNA663902. The proteomic raw (LC-MS/MS) data were deposited in ProteomeXchange via the PRIDE partner repository under the dataset identifier is PXD012412.

## Ethics Statement

The studies involving human participants were reviewed and approved by the Institutional Review Board at Aklilu Lemma Institute of Pathobiology, Addis Ababa University; National Research Ethics Committee of Ethiopia; J. Craig Venter Institute IRB. Written informed consent to participate in this study was provided by the participant or, under the age of 16, his/her legal guardian/next of kin.

## Author Contributions

MH: contributions to all experimental research sections and data analysis. YY: implemented proteomic research and analysis of protein biomarkers and significant manuscript writing contributions. HS: implemented computational part of microbiome analysis and manuscript writing contributions. TTe: contributions to experimental design and biological assays. BW: key contributions to design of human subject study and manuscript review. AW: sample collection and processing. AZ: sample collection and processing. SM: implemented experimental aspects of microbiome analysis using NGS methods. TTs: sample processing. ML: human subject study design. AG: responsible for human subject cohort design and epidemiological aspects and manuscript writing contributions. RP: conceptualized and implemented biomarker study, analyzed and interpreted host-pathogen interaction and immune response data, and wrote all sections of the manuscript. All authors contributed to the article and approved the submitted version.

## Funding

This work was supported by the grant 1U01HG007472 (National Institutes of Health).

## Conflict of Interest

The authors declare that the research was conducted in the absence of any commercial or financial relationships that could be construed as a potential conflict of interest.

## References

[B1] Adami A. J. Cervantes J. L. (2015). The microbiome at the pulmonary alveolar niche and its role in Mycobacterium tuberculosis infection. Tuberculosis (Edinb.) 95, 651–658. 10.1016/j.tube.2015.07.004 26455529 PMC4666774

[B2] Bastian I. Coulter C. National Tuberculosis Advisory C (2017). Position statement on interferon-gamma release assays for the detection of latent tuberculosis infection. Commun. Dis. Intell. Q Rep. 41, E322–EE36. 29864386 10.33321/cdi.2017.41.43

[B3] Bellamy R. Ruwende C. Corrah T. McAdam K. P. Whittle H. C. Hill A. V. (1998). Variations in the NRAMP1 gene and susceptibility to tuberculosis in West Africans. N Engl. J. Med. 338, 640–644. 10.1056/NEJM199803053381002 9486992

[B4] Bellamy R. Ruwende C. Corrah T. McAdam K. P. Thursz M. Whittle H. C.. (1999). Tuberculosis and chronic hepatitis B virus infection in Africans and variation in the vitamin D receptor gene. J. Infect. Dis. 179, 721–724. 10.1086/314614 9952386

[B5] Benarafa C. Priebe G. P. Remold-O’Donnell E. (2007). The neutrophil serine protease inhibitor serpinb1 preserves lung defense functions in Pseudomonas aeruginosa infection. J. Exp. Med. 204, 1901–1909. 10.1084/jem.20070494 17664292 PMC2118684

[B6] Benarafa C. LeCuyer T. E. Baumann M. Stolley J. M. Cremona T. P. Remold-O’Donnell E. (2011). SerpinB1 protects the mature neutrophil reserve in the bone marrow. J. Leukoc. Biol. 90, 21–29. 10.1189/jlb.0810461 21248149 PMC3114599

[B7] Benjamini YH Y. (1995). Controlling the false discovery rate: a practical and powerful approach to multiple testing. J. Roy Statist. Soc. Ser. B 57 1, 289–300. 10.1111/j.2517-6161.1995.tb02031.x

[B8] Berry M. P. Graham C. M. McNab F. W. Xu Z. Bloch S. A. Oni T.. (2010). An interferon-inducible neutrophil-driven blood transcriptional signature in human tuberculosis. Nature 466, 973–977. 10.1038/nature09247 20725040 PMC3492754

[B9] Betlazar C. Middleton R. J. Banati R. Liu G. J. (2020). The Translocator Protein (TSPO) in Mitochondrial Bioenergetics and Immune Processes. Cells 9, 512–529. 10.3390/cells9020512 PMC7072813 32102369

[B10] Bray J. R. C. (1957). J T An ordination of upland forest communities of southern Wisconsin. Ecol. Monogr. 27, 325–349. 10.2307/1942268

[B11] Cao C. Li W. Hua W. Yan F. Zhang H. Huang H.. (2017). Proteomic analysis of sputum reveals novel biomarkers for various presentations of asthma. J. Transl. Med. 15, 171. 10.1186/s12967-017-1264-y 28778200 PMC5544989

[B12] Claassen-Weitz S. Gardner-Lubbe S. Nicol P. Botha G. Mounaud S. Shankar J.. (2018). HIV-exposure, early life feeding practices and delivery mode impacts on faecal bacterial profiles in a South African birth cohort. Sci. Rep. 8, 5078. 10.1038/s41598-018-22244-6 29567959 PMC5864830

[B13] Cox J. Hein M. Y. Luber C. A. Paron I. Nagaraj N. Mann M. (2014). Accurate Proteome-wide Label-free Quantification by Delayed Normalization and Maximal Peptide Ratio Extraction, Termed MaxLFQ. Mol. Cell Proteomics 13, 2513–2526. 10.1074/mcp.M113.031591 24942700 PMC4159666

[B14] De Voss J. J. Rutter K. Schroeder B. G. Su H. Zhu Y. Barry C. E. 3. (2000). The salicylate-derived mycobactin siderophores of Mycobacterium tuberculosis are essential for growth in macrophages. Proc. Natl. Acad. Sci. U. S. A. 97, 1252–1257. 10.1073/pnas.97.3.1252 10655517 PMC15586

[B15] Dewhirst F. E. Chen T. Izard J. Paster B. J. Tanner A. C. Yu W. H.. (2010). The human oral microbiome. J. Bacteriol. 192, 5002–5017. 10.1128/JB.00542-10 20656903 PMC2944498

[B16] Diel R. Goletti D. Ferrara G. Bothamley G. Cirillo D. Kampmann B.. (2011). Interferon-gamma release assays for the diagnosis of latent Mycobacterium tuberculosis infection: a systematic review and meta-analysis. Eur. Respir. J. 37, 88–99. 10.1183/09031936.00115110 21030451

[B17] Dorhoi A. Nouailles G. Jorg S. Hagens K. Heinemann E. Pradl L.. (2012). Activation of the NLRP3 inflammasome by Mycobacterium tuberculosis is uncoupled from susceptibility to active tuberculosis. Eur. J. Immunol. 42, 374–384. 10.1002/eji.201141548 22101787

[B18] Dorresteijn M. J. Visser T. Cox L. A. Bouw M. P. Pillay J. Koenderman A. H.. (2010). C1-esterase inhibitor attenuates the inflammatory response during human endotoxemia. Crit. Care Med. 38, 2139–2145. 10.1097/CCM.0b013e3181f17be4 20693886

[B19] Dye C. Williams B. G. (2010). The population dynamics and control of tuberculosis. Science 328, 856–861. 10.1126/science.1185449 20466923

[B20] Essilfie A. T. Simpson J. L. Dunkley M. L. Morgan L. C. Oliver B. G. Gibson P. G.. (2012). Combined Haemophilus influenzae respiratory infection and allergic airways disease drives chronic infection and features of neutrophilic asthma. Thorax 67, 588–599. 10.1136/thoraxjnl-2011-200160 22387445

[B21] Eum S. Y. Kong J. H. Hong M. S. Lee Y. J. Kim J. H. Hwang S. H.. (2010). Neutrophils are the predominant infected phagocytic cells in the airways of patients with active pulmonary TB. Chest 137 (1), 122–128. 10.1378/chest.09-0903 19749004 PMC2803122

[B22] Fernando S. L. Britton W. J. (2006). Genetic susceptibility to mycobacterial disease in humans. Immunol. Cell Biol. 84, 125–137. 10.1111/j.1440-1711.2006.01420.x 16519730

[B23] Foss C. A. Harper J. S. Wang H. Pomper M. G. Jain S. K. (2013). Noninvasive molecular imaging of tuberculosis-associated inflammation with radioiodinated DPA-713. J. Infect. Dis. 208, 2067–2074. 10.1093/infdis/jit331 23901092 PMC3836460

[B24] Fournier T. Bouach N. Delafosse C. Crestani B. Aubier M. (1999). Inducible expression and regulation of the alpha 1-acid glycoprotein gene by alveolar macrophages: prostaglandin E2 and cyclic AMP act as new positive stimuli. J. Immunol. 163, 2883–2890. 10453035

[B25] Glaser R. Meyer-Hoffert U. Harder J. Cordes J. Wittersheim M. Kobliakova J.. (2009). The antimicrobial protein psoriasin (S100A7) is upregulated in atopic dermatitis and after experimental skin barrier disruption. J. Invest. Dermatol. 129, 641–649. 10.1038/jid.2008.268 18754038

[B26] Goletti D. Petruccioli E. Joosten S. A. Ottenhoff T. H. (2016). Tuberculosis Biomarkers: From Diagnosis to Protection. Infect. Dis. Rep. 8, 6568. 10.4081/idr.2016.6568 27403267 PMC4927936

[B27] Gopal R. Lin Y. Obermajer N. Slight S. Nuthalapati N. Ahmed M.. (2012). IL-23-dependent IL-17 drives Th1-cell responses following Mycobacterium bovis BCG vaccination. Eur. J. Immunol. 42, 364–373. 10.1002/eji.201141569 22101830 PMC3490408

[B28] Gopal R. Monin L. Torres D. Slight S. Mehra S. McKenna K. C.. (2013). S100A8/A9 proteins mediate neutrophilic inflammation and lung pathology during tuberculosis. Am. J. Respir. Crit. Care Med. 188, 1137–1146. 10.1164/rccm.201304-0803OC 24047412 PMC3863739

[B29] Gopal R. Monin L. Slight S. Uche U. Blanchard E. Fallert Junecko B. A.. (2014). Unexpected role for IL-17 in protective immunity against hypervirulent Mycobacterium tuberculosis HN878 infection. PloS Pathog. 10, e1004099. 10.1371/journal.ppat.1004099 24831696 PMC4022785

[B30] Gould J. M. Weiser J. N. (2001). Expression of C-reactive protein in the human respiratory tract. Infect. Immun. 69, 1747–1754. 10.1128/IAI.69.3.1747-1754.2001 11179352 PMC98081

[B31] Grassl N. Kulak N. A. Pichler G. Geyer P. E. Jung J. Schubert S.. (2016). Ultra-deep and quantitative saliva proteome reveals dynamics of the oral microbiome. Genome Med. 8, 44. 10.1186/s13073-016-0293-0 27102203 PMC4841045

[B32] HaileMariam M. Eguez R. V. Singh H. Bekele S. Ameni G. Pieper R.. (2018). S-Trap, an Ultrafast Sample-Preparation Approach for Shotgun Proteomics. J. Proteome Res. 17, 2917–2924. 10.1021/acs.jproteome.8b00505 30114372

[B33] Hardwick M. J. Chen M. K. Baidoo K. Pomper M. G. Guilarte T. R. (2005). In vivo imaging of peripheral benzodiazepine receptors in mouse lungs: a biomarker of inflammation. Mol. Imaging 4, 432–438. 10.2310/7290.2005.05133 16285905

[B34] Hasty K. A. Pourmotabbed T. F. Goldberg G. I. Thompson J. P. Spinella D. G. Stevens R. M.. (1990). Human Neutrophil Collagenase - a Distinct Gene-Product with Homology to Other Matrix Metalloproteinases. J. Biol. Chem. 265, 11421–11424. 10.1016/S0021-9258(19)38413-3 2164002

[B35] Hermans C. Bernard A. (1999). Lung epithelium-specific proteins: characteristics and potential applications as markers. Am. J. Respir. Crit. Care Med. 159, 646–678. 10.1164/ajrccm.159.2.9806064 9927386

[B36] Hong B. Y. Maulen N. P. Adami A. J. Granados H. Balcells M. E. Cervantes J. (2016). Microbiome Changes during Tuberculosis and Antituberculous Therapy. Clin. Microbiol. Rev. 29, 915–926. 10.1128/CMR.00096-15 27608937 PMC5010754

[B37] Inoue M. Niki M. Ozeki Y. Nagi S. Chadeka E. A. Yamaguchi T.. (2018). High-density lipoprotein suppresses tumor necrosis factor alpha production by mycobacteria-infected human macrophages. Sci. Rep. 8, 6736. 10.1038/s41598-018-24233-1 29712918 PMC5928146

[B38] Jiang J. Wang X. Wang X. Cao Z. Liu Y. Dong M.. (2010). Reduced CD27 expression on antigen-specific CD4+ T cells correlates with persistent active tuberculosis. J. Clin. Immunol. 30, 566–573. 10.1007/s10875-010-9418-1 20393787

[B39] Jindal S. K. Jindal A. Agarwal R. (2016). Upper Respiratory Tract Tuberculosis. Microbiol. Spectr. 4, 1–9. 10.1128/microbiolspec.TNMI7-0009-2016 27837744

[B40] Kell D. B. Pretorius E. (2014). Serum ferritin is an important inflammatory disease marker, as it is mainly a leakage product from damaged cells. Metallomics 6, 748–773. 10.1039/C3MT00347G 24549403

[B41] Kim J. Wessling-Resnick M. (2012). The Role of Iron Metabolism in Lung Inflammation and Injury. J. Allergy Ther. 3 (Suppl 4), 4. 10.4172/2155-6121.S4-004 PMC5718378 29226014

[B42] Kim H. S. Lyons K. M. Saitoh E. Azen E. A. Smithies O. Maeda N. (1993). The structure and evolution of the human salivary proline-rich protein gene family. Mamm. Genome. 4, 3–14. 10.1007/BF00364656 8422499

[B43] Kurthkoti K. Amin H. Marakalala M. J. Ghanny S. Subbian S. Sakatos A.. (2017). The Capacity of Mycobacterium tuberculosis To Survive Iron Starvation Might Enable It To Persist in Iron-Deprived Microenvironments of Human Granulomas. mBio 8 (4), e01092–17. 10.1128/mBio.01092-17 PMC5559634 28811344

[B44] Love M. I. Huber W. Anders S. (2014). Moderated estimation of fold change and dispersion for RNA-seq data with DESeq2. Genome Biol. 15, 550. 10.1186/s13059-014-0550-8 25516281 PMC4302049

[B45] Lowe D. M. Redford P. S. Wilkinson R. J. O’Garra A. Martineau A. R. (2012). Neutrophils in tuberculosis: friend or foe? Trends Immunol. 33, 14–25. 10.1016/j.it.2011.10.003 22094048

[B46] Lu L. Li J. Moussaoui M. Boix E. (2018). Immune Modulation by Human Secreted RNases at the Extracellular Space. Front. Immunol. 9, 1012. 10.3389/fimmu.2018.01012 29867984 PMC5964141

[B47] Machingaidze S. Wiysonge C. S. Gonzalez-Angulo Y. Hatherill M. Moyo S. Hanekom W.. (2011). The utility of an interferon gamma release assay for diagnosis of latent tuberculosis infection and disease in children: a systematic review and meta-analysis. Pediatr. Infect. Dis. J. 30, 694–700. 10.1097/INF.0b013e318214b915 21427627

[B48] Martinez Cordero E. Gonzalez M. M. Aguilar L. D. Orozco E. H. Hernandez Pando R. (2008). Alpha-1-acid glycoprotein, its local production and immunopathological participation in experimental pulmonary tuberculosis. Tuberculosis (Edinb.) 88, 203–211. 10.1016/j.tube.2007.10.004 18055265

[B49] McMurdie P. J. Holmes S. (2013). phyloseq: an R package for reproducible interactive analysis and graphics of microbiome census data. PloS One 8, e61217. 10.1371/journal.pone.0061217 23630581 PMC3632530

[B50] McMurdie P. J. Holmes S. (2014). Waste not, want not: why rarefying microbiome data is inadmissible. PloS Comput. Biol. 10, e1003531. 10.1371/journal.pcbi.1003531 24699258 PMC3974642

[B51] Miranda P. Gil-Santana L. Oliveira M. G. Mesquita E. D. Silva E. Rauwerdink A.. (2017). Sustained elevated levels of C-reactive protein and ferritin in pulmonary tuberculosis patients remaining culture positive upon treatment initiation. PLoS One 12, e0175278. 10.1371/journal.pone.0175278 28384354 PMC5383283

[B52] Naidoo C. C. Nyawo G. R. Wu B. G. Walzl G. Warren R. M. Segal L. N.. (2019). The microbiome and tuberculosis: state of the art, potential applications, and defining the clinical research agenda. Lancet Respir. Med. 7, 892–906. 10.1016/S2213-2600(18)30501-0 30910543

[B53] Parrow N. L. Fleming R. E. Minnick M. F. (2013). Sequestration and scavenging of iron in infection. Infect. Immun. 81, 3503–3514. 10.1128/IAI.00602-13 23836822 PMC3811770

[B54] Perry S. de Jong B. C. Solnick J. V. de la Luz Sanchez M. Yang S. Lin P. L.. (2010). Infection with Helicobacter pylori is associated with protection against tuberculosis. PLoS One 5, e8804. 10.1371/journal.pone.0008804 20098711 PMC2808360

[B55] Potian J. A. Rafi W. Bhatt K. McBride A. Gause W. C. Salgame P. (2011). Preexisting helminth infection induces inhibition of innate pulmonary anti-tuberculosis defense by engaging the IL-4 receptor pathway. J. Exp. Med. 208, 1863–1874. 10.1084/jem.20091473 21825018 PMC3171086

[B56] Pucino V. Bombardieri M. Pitzalis C. Mauro C. (2017). Lactate at the crossroads of metabolism, inflammation, and autoimmunity. Eur. J. Immunol. 47, 14–21. 10.1002/eji.201646477 27883186

[B57] Quast C. Pruesse E. Yilmaz P. Gerken J. Schweer T. Yarza P.. (2013). The SILVA ribosomal RNA gene database project: improved data processing and web-based tools. Nucleic Acids Res. 41, D590–D596. 10.1093/nar/gks1219 23193283 PMC3531112

[B58] Raviglione M. C. Snider D. E. Jr. Kochi A. (1995). Global epidemiology of tuberculosis. Morbidity and mortality of a worldwide epidemic. JAMA 273, 220–226. 10.1001/jama.1995.03520270054031 7807661

[B59] Read C. B. Kuijper J. L. Hjorth S. A. Heipel M. D. Tang X. Fleetwood A. J.. (2015). Cutting Edge: identification of neutrophil PGLYRP1 as a ligand for TREM-1. J. Immunol. 194, 1417–1421. 10.4049/jimmunol.1402303 25595774 PMC4319313

[B60] Ryu Y. J. (2015). Diagnosis of pulmonary tuberculosis: recent advances and diagnostic algorithms. Tuberc. Respir. Dis. (Seoul) 78, 64–71. 10.4046/trd.2015.78.2.64 25861338 PMC4388902

[B61] Sagel S. D. Chmiel J. F. Konstan M. W. (2007). Sputum biomarkers of inflammation in cystic fibrosis lung disease. Proc. Am. Thorac. Soc 4, 406–417. 10.1513/pats.200703-044BR 17652508 PMC2647605

[B62] Sala C. Benjak A. Goletti D. Banu S. Mazza-Stadler J. Jaton K.. (2020). Multicenter analysis of sputum microbiota in tuberculosis patients. PLoS One 15 (10), e0240250. 10.1371/journal.pone.0240250 33044973 PMC7549818

[B63] Sandhu G. Battaglia F. Ely B. K. Athanasakis D. Montoya R. Valencia T.. (2012). Discriminating active from latent tuberculosis in patients presenting to community clinics. PLoS One 7, e38080. 10.1371/journal.pone.0038080 22666453 PMC3364185

[B64] Schleef R. R. Chuang T. L. (2000). Protease inhibitor 10 inhibits tumor necrosis factor alpha -induced cell death. Evidence for the formation of intracellular high M(r) protease inhibitor 10-containing complexes. J. Biol. Chem. 275, 26385–26389. 10.1074/jbc.C000389200 10871600

[B65] Schloss P. D. Westcott S. L. Ryabin T. Hall J. R. Hartmann M. Hollister E. B.. (2009). Introducing mothur: open-source, platform-independent, community-supported software for describing and comparing microbial communities. Appl. Environ. Microbiol. 75, 7537–7541. 10.1128/AEM.01541-09 19801464 PMC2786419

[B66] Singh H. Torralba M. G. Moncera K. J. DiLello L. Petrini J. Nelson K. E.. (2019). Gastro-intestinal and oral microbiome signatures associated with healthy aging. Geroscience 41, 907–921. 10.1007/s11357-019-00098-8 31620923 PMC6925087

[B67] Smith I. (2003). Mycobacterium tuberculosis pathogenesis and molecular determinants of virulence. Clin. Microbiol. Rev. 16, 463–496. 10.1128/CMR.16.3.463-496.2003 12857778 PMC164219

[B68] Soborg C. Madsen H. O. Andersen A. B. Lillebaek T. Kok-Jensen A. Garred P. (2003). Mannose-binding lectin polymorphisms in clinical tuberculosis. J. Infect. Dis. 188, 777–782. 10.1086/377183 12934195

[B69] Song S. H. Han M. Choi Y. S. Dan K. S. Yang M. G. Song J.. (2014). Proteomic profiling of serum from patients with tuberculosis. Ann. Lab. Med. 34, 345–353. 10.3343/alm.2014.34.5.345 25187886 PMC4151002

[B70] Teklu T. Kwon K. Wondale B. HaileMariam M. Zewude A. Medhin G.. (2018a). Potential Immunological Biomarkers for Detection of Mycobacterium tuberculosis Infection in a Setting Where M. tuberculosis Is Endemic, Ethiopia. Infect. Immun. 86 (4), e00759–7, 1–11. 10.1128/IAI.00759-17 PMC5865051 29311240

[B71] Teklu T. Legesse M. Medhin G. Zewude A. Chanyalew M. Zewdie M.. (2018b). Latent tuberculosis infection and associated risk indicators in pastoral communities in southern Ethiopia: a community based cross-sectional study. BMC Public Health 18, 266. 10.1186/s12889-018-5149-7 29454325 PMC5816385

[B72] Teo S. M. Mok D. Pham K. Kusel M. Serralha M. Troy N.. (2015). The infant nasopharyngeal microbiome impacts severity of lower respiratory infection and risk of asthma development. Cell Host Microbe 17, 704–715. 10.1016/j.chom.2015.03.008 25865368 PMC4433433

[B73] Torrado E. Cooper A. M. (2010). IL-17 and Th17 cells in tuberculosis. Cytokine Growth Factor Rev. 21, 455–462. 10.1016/j.cytogfr.2010.10.004 21075039 PMC3032416

[B74] Tufariello J. M. Chan J. Flynn J. L. (2003). Latent tuberculosis: mechanisms of host and bacillus that contribute to persistent infection. Lancet Infect. Dis. 3, 578–590. 10.1016/S1473-3099(03)00741-2 12954564

[B75] Uhlen M. Fagerberg L. Hallstrom B. M. Lindskog C. Oksvold P. Mardinoglu A.. (2015). Proteomics. Tissue-based map of the human proteome. Science 347, 1260419. 10.1126/science.1260419 25613900

[B76] Uranga C. C. Arroyo P. Jr. Duggan B. M. Gerwick W. H. Edlund A. (2020). Commensal Oral Rothia mucilaginosa Produces Enterobactin, a Metal-Chelating Siderophore. mSystems 5 27 (2), e00161–20 10.1128/mSystems.00161-20 PMC7190385 32345739

[B77] Vareille M. Kieninger E. Edwards M. R. Regamey N. (2011). The airway epithelium: soldier in the fight against respiratory viruses. Clin. Microbiol. Rev. 24, 210–229. 10.1128/CMR.00014-10 21233513 PMC3021210

[B78] Welch B. L. (1947). The generalisation of student’s problems when several different population variances are involved. Biometrika 34, 28–35. 10.1093/biomet/34.1-2.28 20287819

[B79] Wondale B. Medihn G. Teklu T. Mersha W. Tamirat M. Ameni G. (2017). A retrospective study on tuberculosis treatment outcomes at Jinka General Hospital, southern Ethiopia. BMC Res. Notes. 10, 680. 10.1186/s13104-017-3020-z 29202880 PMC5715540

[B80] Wondale B. Medhin G. Abebe G. Tolosa S. Mohammed T. Teklu T.. (2018). Phenotypic and genotypic drug sensitivity of Mycobacterium tuberculosis complex isolated from South Omo Zone, Southern Ethiopia. Infect. Drug Resist. 11, 1581–1589. 10.2147/IDR.S165088 30288068 PMC6161742

[B81] Wondale B. Keehwan K. Medhin G. Teklu T. Mohammed T. Tolosa S.. (2020). Molecular epidemiology of clinical Mycobacterium tuberculosis complex isolates in South Omo, Southern Ethiopia. BMC Infect. Dis. 13, 750–761. 10.1186/s12879-020-05394-9 PMC7557052 33050903

[B82] World Health Organization (1997). Tuberculosis control: The DOTS Strategy (Directly Observed Treatment Short-Course). Ed. Organization W. H. (Geneva, Switzerland: World Health Organization), 14.

[B83] World Health Organization (2015). Use of high burden country lists for TB by WHO in the post-2015 era (Geneva, Switzerland: World Health Organization, Meeting of WHO’s Strategic and Technical Advisory Group for TB).

[B84] Wu J. Liu W. He L. Huang F. Chen J. Cui P.. (2013). Sputum microbiota associated with new, recurrent and treatment failure tuberculosis. PLoS One 8, e83445. 10.1371/journal.pone.0083445 24349510 PMC3862690

[B85] Wu C. C. Chu H. W. Hsu C. W. Chang K. P. Liu H. P. (2015). Saliva proteome profiling reveals potential salivary biomarkers for detection of oral cavity squamous cell carcinoma. Proteomics 15, 3394–3404. 10.1002/pmic.201500157 26205615

[B86] Xu D. Pavlidis P. Thamadilok S. Redwood E. Fox S. Blekhman R.. (2016). Recent evolution of the salivary mucin MUC7. Sci. Rep. 6, 31791. 10.1038/srep31791 27558399 PMC4997351

[B87] Yu Y. Smith M. Pieper R. (2014a). A spinnable and automatable StageTip for high throughput peptide desalting and proteomics. Protocol Exchange. 10.1038/protex.2014.033

[B88] Yu Y. Suh M.-J. Sikorski P. Kwon K. Nelson K. E. Pieper R. (2014b). Urine Sample Preparation in 96-Well Filter Plates for Quantitative Clinical Proteomics. Anal. Chem. 86, 5470–5477. 10.1021/ac5008317 24797144 PMC4045327

[B89] Yu Y. Sikorski P. Smith M. Bowman-Gholston C. Cacciabeve N. Nelson K. E.. (2017a). Comprehensive Metaproteomic Analyses of Urine in the Presence and Absence of Neutrophil-Associated Inflammation in the Urinary Tract. Theranostics 7, 238–252. 10.7150/thno.16086 28042331 PMC5197061

[B90] Yu Y. Kwon K. Tsitrin T. Bekele S. Sikorski P. Nelson K. E.. (2017b). Characterization of Early-Phase Neutrophil Extracellular Traps in Urinary Tract Infections. PLoS Pathog. 13, e1006151. 10.1371/journal.ppat.1006151 28129394 PMC5298345

[B91] Zhang X. Liu F. Li Q. Jia H. Pan L. Xing A.. (2014). A proteomics approach to the identification of plasma biomarkers for latent tuberculosis infection. Diagn. Microbiol. Infect. Dis. 79, 432–437. 10.1016/j.diagmicrobio.2014.04.005 24865408 PMC7127109

