# Supplementary material for: Protein and Microbial Biomarkers in Sputum Discern Acute and Latent Tuberculosis in Investigation of Pastoral Ethiopian Cohort
Source: Front Cell Infect Microbiol. 2021 Jun 4;11:595554. doi: 10.3389/fcimb.2021.595554 (PMC8212885; doi:10.3389/fcimb.2021.595554)
Supplement: Supplementary file 3 [file Presentation_1.pdf]

Supplementary Materials (Supplementary Presentation 1)

A. Display of correlation coefficients (R) for LC-MS/MS proteomes

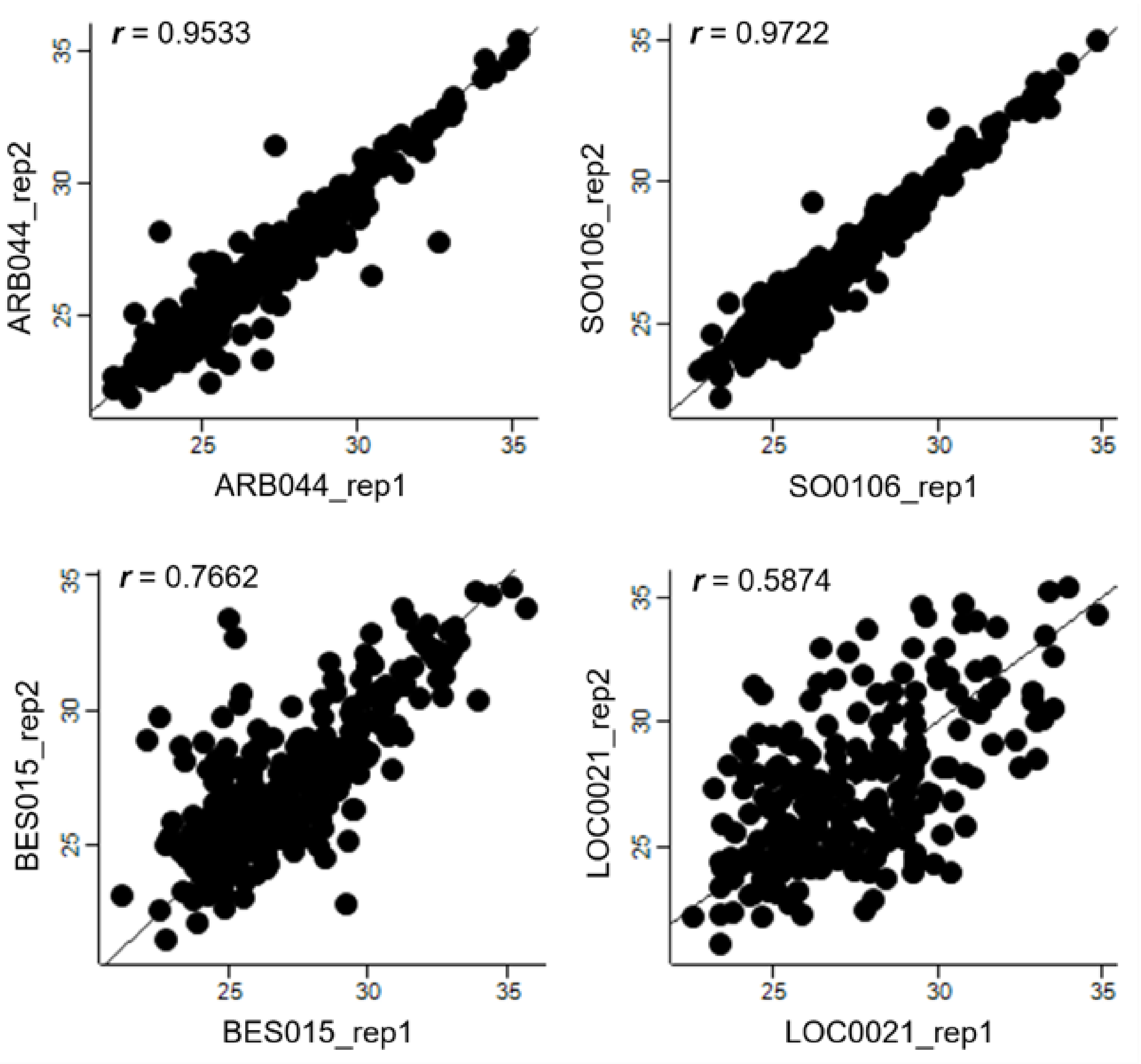

Top panel: Correlation coefficients for protein abundances from LC-MS/MS technical replicates. Bottom panel: Correlation coefficients for protein abundances from LC-MS/MS data of biologically distinct samples (2 cases each). R-values are based on 432 quality-filtered and quantified sputum proteins in the sputum proteome.

B. Pearson correlation hierarchical clustering analysis of 75 sputum proteomic datasets

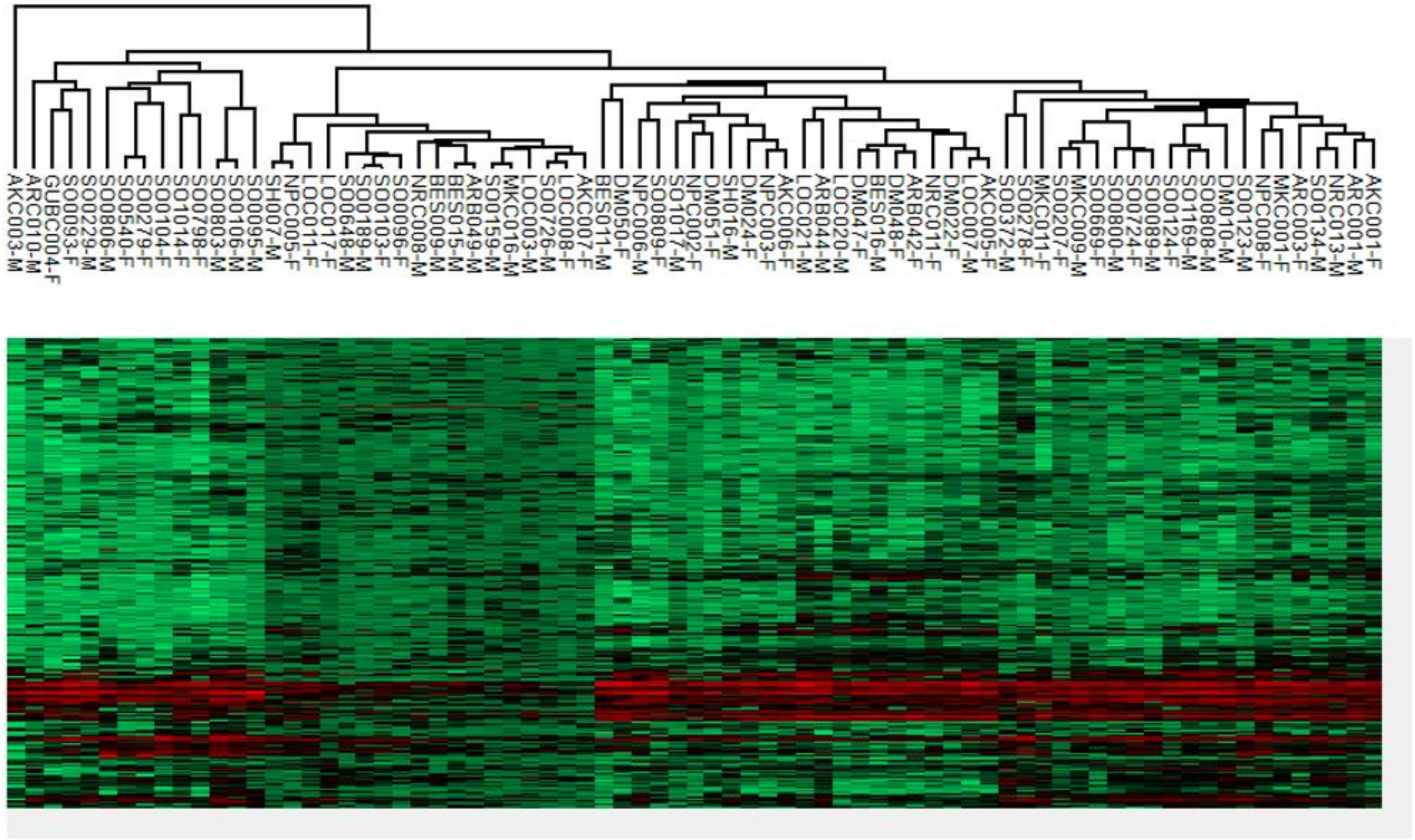

Using 432 LFQ-quantified proteins, specimens were clustered using the Pearson Correlation to assess clustering of (1) female (F) and male (M) specimens and (2) the biological groups: the pulmonary tuberculosis (PTB) group identifiers start with “SO”, the latent TB infection (LTBI) and negative community control (NCC) group identifiers start with other letters. The heat map illustrates protein abundances from red (high) to back (medium) and green (low).

C. Western blots of α-1-acid glycoprotein in sputum analyzing seven TB, six LTBI and five NCC samples.

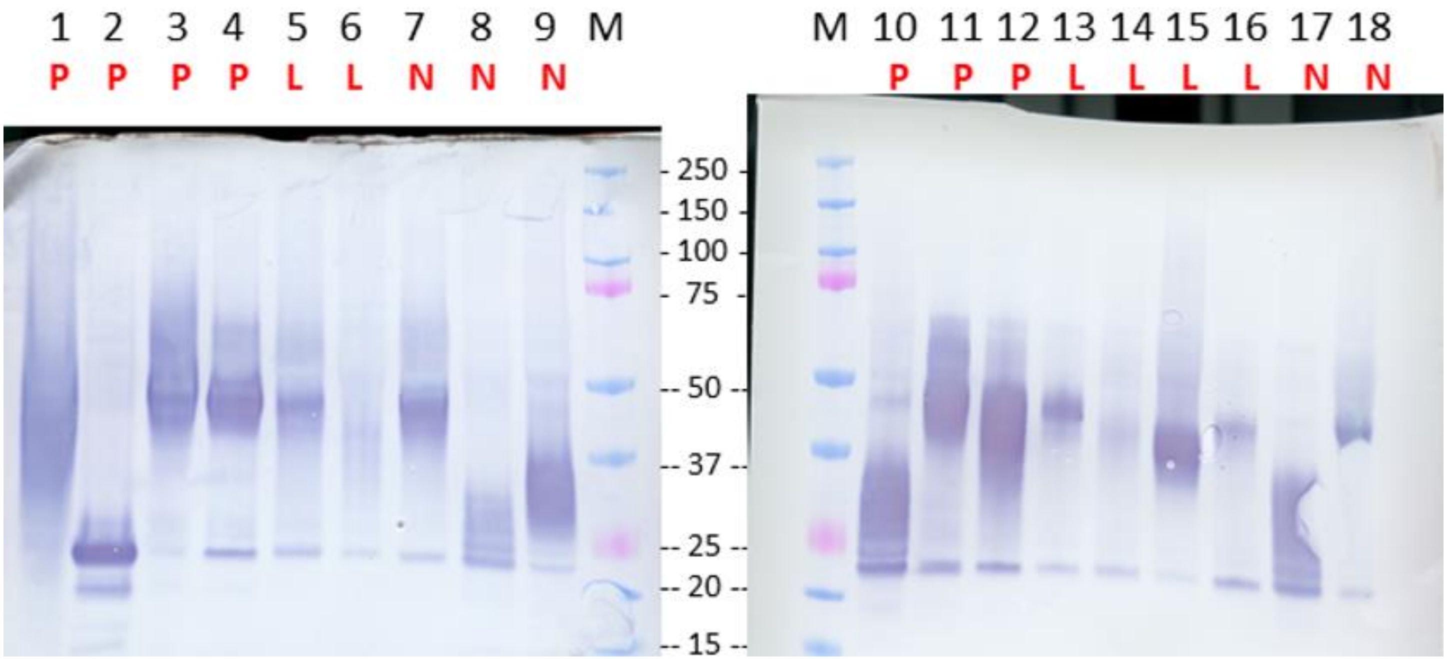

Western blots of α-1-acid glycoprotein using a primary anti-human α-1-acid glycoprotein (α1-AGP) polyclonal antibody (IgG fraction) developed in rabbit (Sigma-Aldrich; A0534) was used at a 1:2,000 dilution. Sputum samples correspond to seven PTB (denoted **P**), six LTBI (denoted **L**) and five NCC (denoted **N**) subjects as denoted in the blot images. Separation in 4-12%T SDS-PAGE gels was followed by western blotting visualizing α1-AGP with 3,3'-diaminobenzidine. M: molecular weight markers, with numbers equaling size in kiloDalton. Due to variable glycosylation and likely proteolytic processing, α1-AGP is stained in a wide Mr range.

D. α-Diversity analysis of the sputum microbiomes using Chao1 and Shannon index calculations

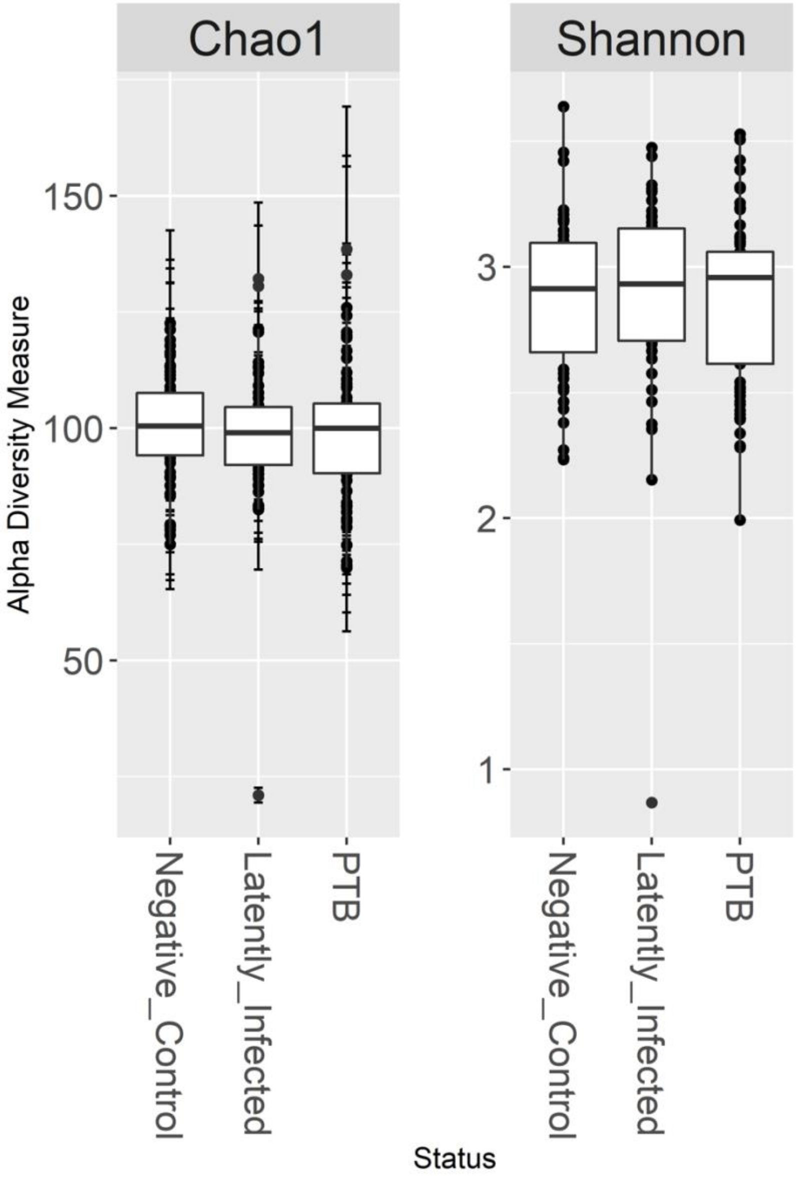

α-diversity calculations are based on 16S rRNA phylogenetic profiling using V4 region amplicons. Compared were pulmonary tuberculosis (PTB) subjects, latently infected subjects (LTBI), and negative community controls. There were no statistically significant differences in α-diversity using either the Chao1 or Shannon method.

E. **Principle component analysis of sputum microbiome datasets for pulmonary TB (PTB), latently infected, and negative community controls.**

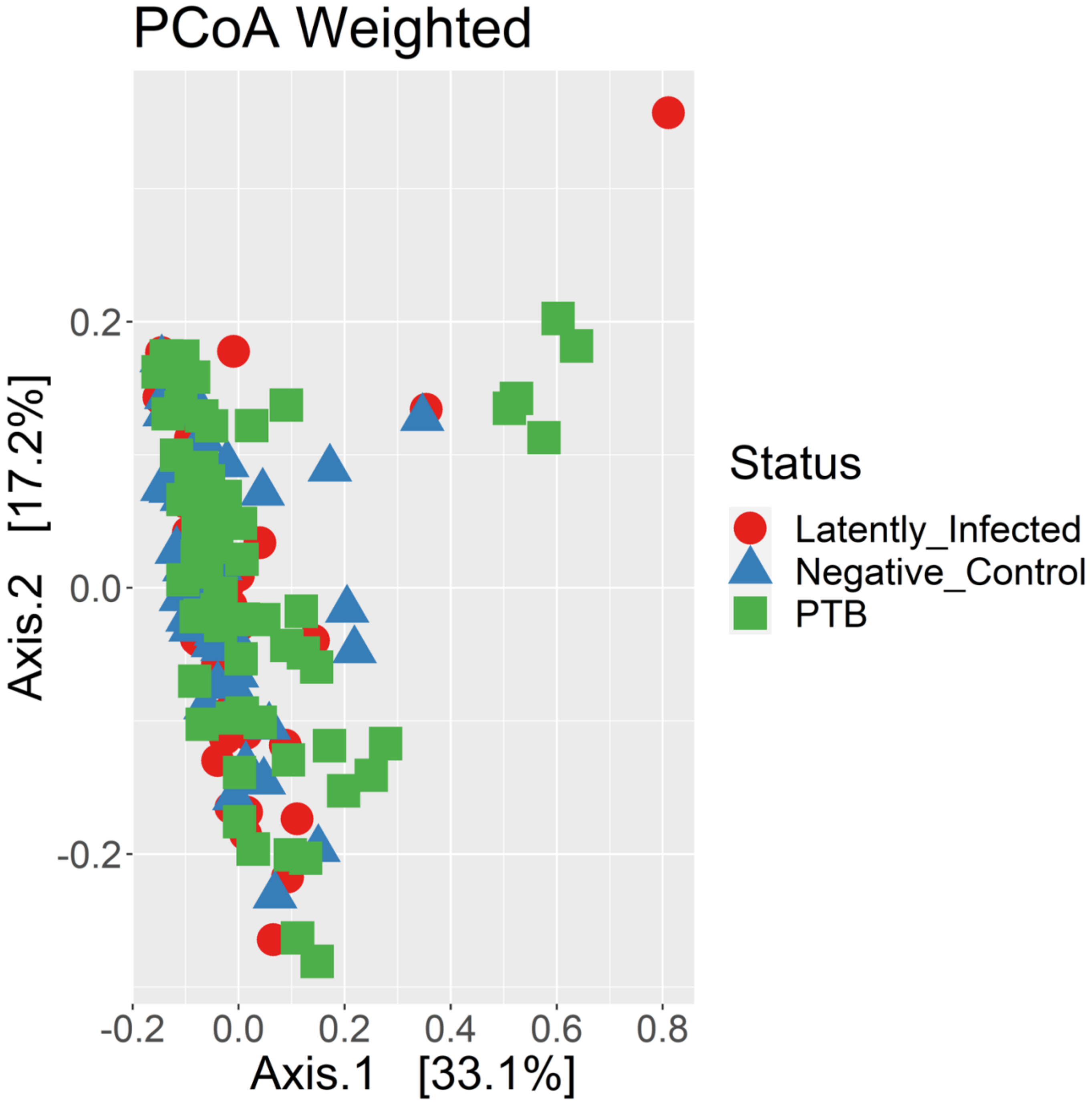

Principle component analysis (PCoA) displayed with two Principal Components explaining most of the variance between the three datasets. The data are based on phylogenetic taxonomy assignments at the genus level based on bacterial 16S rRNA V4 region amplicons (MiSeq multiplexed sequencing). Compared were pulmonary tuberculosis (PTB) subjects, latently infected subjects (LTBI), and negative community controls. There was no clear separation of microbial profiles comparing the groups.

F. 16S rRNA analysis reveals presence of genus *Mycobacterium* in only PTB specimens supporting detectable levels of *M. tuberculosis*

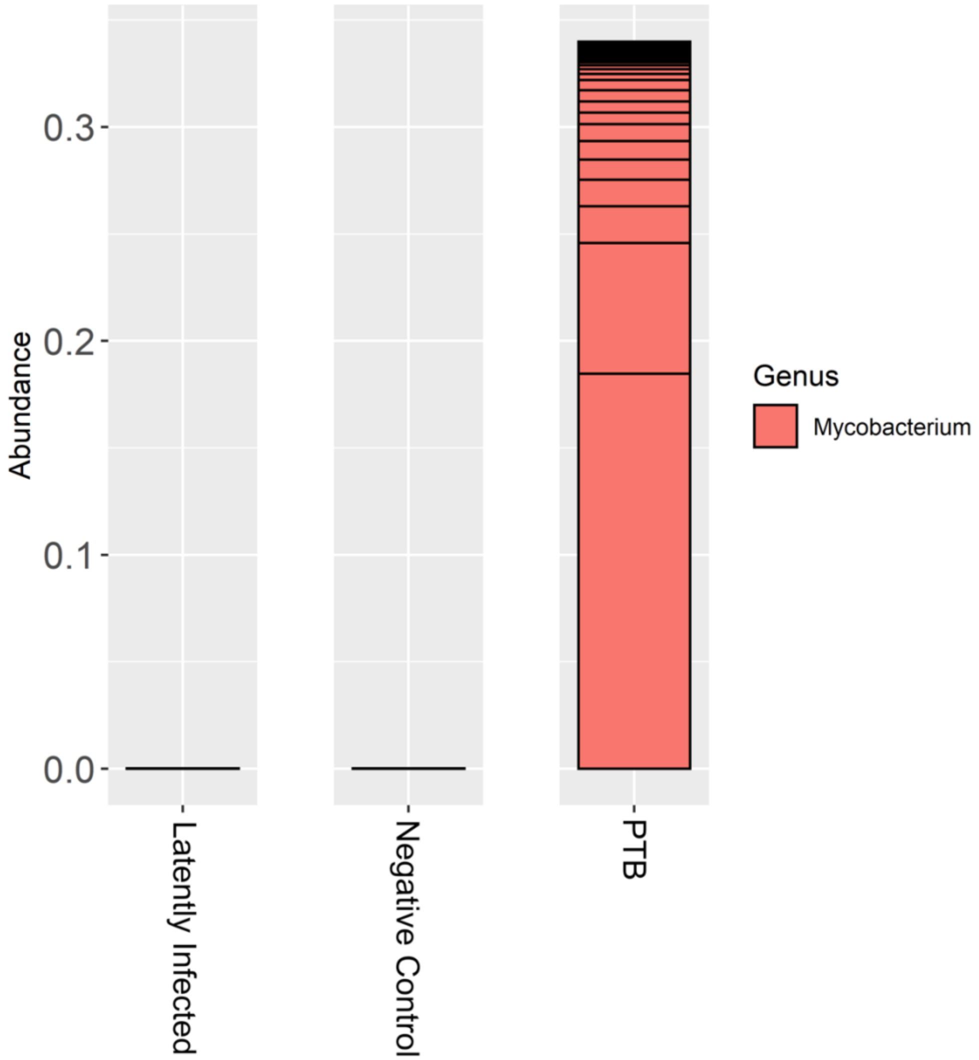

16S rRNA phylogenetic profiling was based on V4 region amplicons. The 16S rRNA sequence based on operational taxonomic unit assignment to the genus Mycobacterium was limited to sputum extracts of pulmonary tuberculosis (PTB) specimens.
